# A portfolio selection model based on the knapsack problem under uncertainty

**DOI:** 10.1371/journal.pone.0213652

**Published:** 2019-05-01

**Authors:** Fereshteh Vaezi, Seyed Jafar Sadjadi, Ahmad Makui

**Affiliations:** 1 Department of Industrial Engineering, Iran University of Science and Technology, Narmak, Tehran, Iran; 2 Department of Industrial Engineering, Iran University of Science and Technology, Narmak, Tehran, Iran; 3 Department of Industrial Engineering, Iran University of Science and Technology, Narmak, Tehran, Iran; Beihang University, CHINA

## Abstract

One of the primary concerns in investment planning is to determine the number of shares for asset with relatively high net value of share such as Berkshire Hathaway on Stock market. Traditional asset allocation methods like Markowitz theorem gives the solution as a percentage and this ratio may suggest allocation of half of a share on the market, which is impractical. Thus, it is necessary to propose a method to determine the number of shares for each asset. This paper presents a knapsack based portfolio selection model where the expected returns, prices, and budget are characterized by interval values. The study determines the priority and importance of each share in the proposed model by extracting the interval weights from an interval comparison matrix. The resulted model is converted into a parametric linear programming model in which the decision maker is able to determine the optimism threshold. Finally, a discrete firefly algorithm is designed to find the near optional solutions in large dimensions. The proposed study is implemented for some data from the US stock exchange.

## Introduction

Markowitz [1,2] introduced a mean-variance model to select the optimal asset portfolio. The main task of the portfolio selection model is to allocate capital among different assets such that the risk and return are simultaneously optimized in the portfolio. Markowitz model is based on some assumptions that are rarely feasible in reality. Hence, there has been widespread efforts by many researchers to provide methods for analyzing stocks in financial markets and improving these methods in the world (see [3]).

The standard mean-variance Markowitz model does not consider the real financial market constraints, including transaction costs [4,5], Cardinality Constraint [6,7], and multi-period Cardinality Constraint Mean-Variance (CCMV) [8–10].

Another problem in the real financial market is that decision-making processes often face with complexity and uncertainty. So, many approaches based on the uncertain conditions have been developed to consider the real condition of financial markets in portfolio selection models, including, a robust-based approach in [11], a scenario-based approach in [12,13], and fuzzy methods in [14,15]. Fuzzy sets was introduced by Zadeh [16] and interval-valued fuzzy sets was presented by Turksen [17]. During the past few years, a number of studies have examined the interval fuzzy uncertainty for the mean-variance Markowitz model [18–20]. The use of interval approaches converts the model into a nonlinear model, therefore, Liu [21] proposed two linear programming approaches to obtain the upper and lower bounds of the interval number of returns in the Markowitz model. In addition, a multi-period MV model with the interval returns, risk and turnover rates of risky assets was proposed by Liu et al. [22,23]. When all or part of the quantitative and accurate data is not available, some qualitative portfolio models under the fuzzy environment was proposed by Zhou and Xu [24].

On the other hand in the Markowitz model, the variance is considered as a risk measurement factor. Variance is known as a symmetric risk measurement factor and has been criticized by many researchers. Thus, other studies also used different risk measures in the portfolio optimization model and then examined the model under interval uncertainty. For example, mean-Value-at-Risk (VAR) portfolio optimization model [25], Mean-Variance-Skewness (MVS) portfolio selection model [26], and mean-semi-absolute deviation portfolio selection model [27–29] are some of the examples which have examined the portfolio optimization under interval uncertainty.

Moreover, note that the structure of the decision variable in the mean-variance Markowitz model is a continuous variable. One of particular interest is the finite divisibility of the (stock) assets, i.e. the obligation to buy/sell only integer quantities of asset lots whose number is pre-established. In some cases, we need to properly allocate the number of shares to different assets. Thus, Li et al. [30] introduced a hybrid algorithm based on a convergent Lagrangian and a contour-domain cut method for solving a nonlinear integer portfolio optimization problem with concave transaction costs and cardinality constraint. Corazza and Favaretto [31] presented a hybrid algorithm based on the relaxation method and two rounding approaches for quadratic mixed-integer programming problem based on one riskless asset and *n* risky assets. Li and Tsai [32] presented a hybrid algorithm based on the dual Lagrangian relaxation and transformation approaches to examine a portfolio selection model with a nonlinear objective function, transaction costs, and integer variables. Bonami and Lejeune [33] proposed a non-linear branch-and-bound algorithm based on a stochastic branching and an integrated dynamic branching for portfolio optimization with integer variables. Anagnostopoulos and Mamanis [34] compared different multi-objective evolutionary algorithms to study a nonlinear mixed-integer three-objective problem with class and quantity limitations. Castro et al. [35] proposed a mathematical algorithm based on various test sets to solve a portfolio selection model with a nonlinear constraint and integer variable.

Therefore, it is very important to provide portfolio selection models that can properly allocate the number of shares to different assets. Therefore, in this research, a portfolio selection model based on the knapsack problem with the risk preferences of investors, cardinality constraint, floor and ceiling constraint, and budget constraint is presented. Since it is far from the fact that information data are considered accurate and definitive, all information or part of them is handled using the fuzzy sets or interval values. In this paper, the interval-valued fuzzy sets are used to examine a portfolio optimization problem under uncertain conditions. Also, a discrete firefly algorithm is designed for the solution. A numerical example of the US stock exchange is given to illustrate the application of the proposed model and demonstrates the effectiveness of the designed algorithm for solving the proposed model.

The structure of this paper is as follows. In section 2, some preliminary notions such as the interval numbers, interval comparison matrix, and interval weights are defined. In Section 3, the knapsack based portfolio optimization model considering the risk preferences of investors under interval uncertainty is presented. In Section 4, Discrete Firefly Algorithm (DFA) is presented for the proposed optimization model. A numerical example and sensitivity analysis are examined in Section 5. Finally, a brief summary of the paper, some conclusions, and some future studies are given in Section 6.

## Preliminaries

In this section, a review of some necessary concepts is presented:

### Interval numbers

Interval is represented as *A* = 〈*m*(*A*), *w*(*A*)〉 where:

$$m(A)=\frac{1}{2}(\underline{a}+\bar{a})\;\text{and}\;w(A)=\frac{1}{2}(\bar{a}-\underline{a}).$$ (1)

Alefeld and Herzberger [36,37] presented the mathematical operators (〈*OP*〉 ∈ {±,⊗}) between interval numbers $A=[\underline{a},\bar{a}]\;$ and $B=[\underline{b},\bar{b}]$ as follows:

$$[\underline{a},\bar{a}]\langle OP\rangle [\underline{b},\bar{b}]={\left[\begin{array}{l} \text{min}\left(\left(\underline{a}\langle OP\rangle \underline{b}\right),\left(\underline{a}\;\langle OP\rangle \bar{b}\right),\left(\bar{a}\langle OP\rangle \underline{b}\right),\left(\bar{a}\langle OP\rangle \bar{b}\right)\right),\\ \text{max}\left(\left(\underline{a}\;\langle OP\rangle \underline{b}\right),\left(\underline{a}\;\langle OP\rangle \bar{b}\right),\left(\bar{a}\langle OP\rangle \underline{b}\right),\left(\bar{a}\langle OP\rangle \bar{b}\right)\right) \end{array}\right]}^{t}.$$ (2)

In addition, the multiplication operator (⊗) between number *λ* (λ∈ℜ) and interval number $\;A=[\underline{a},\bar{a}]\;$ is defined as follows:

$$\lambda ⊗A=\lambda ⊗[\underline{a},\bar{a}]=\{\begin{array}{ll} \left[\lambda \underline{a},\lambda \bar{a}\right] & if\lambda \geq 0\\ \left[\lambda \bar{a},\lambda \underline{a}\right] & f\lambda \leq 0 \end{array}.$$ (3)

Also, the division operator between interval numbers $\;A=[\underline{a},\bar{a}]\;$ and $\;B=[\underline{b},\bar{b}]\;$ is as follows:

$$[\underline{a}\;,\;\bar{a}]/[\underline{b}\;,\;\bar{b}]=[\underline{a}/\bar{b},\bar{a}/\underline{b}].$$ (4)

More related information of the interval numbers can be found in [38].

### Interval comparison matrix and interval weights

Wang and Elhag [39] introduced a method for extracting interval weights from an interval comparison matrix. In this method, an interval comparison matrix is considered as follows:

$$A=\left[\begin{array}{cccc} 1 & [l_{12},\;u_{12}] & \cdots & [l_{1n},\;u_{1n}]\\ {}[l_{21},\;u_{21}] & 1 & \cdots & [l_{2n},\;u_{2n}]\\ \vdots & \vdots & \cdots & \vdots \\ {}[l_{n1},\;u_{n1}] & [l_{n2},\;u_{n2}] & \cdots & 1 \end{array}\right],$$ (5)

where *l*_*ij*_ ≥ 0, *l*_*ij*_ ≤ *u*_*ij*_, *l*_*ij*_ = 1/*u*_*ij*_, and *u*_*ij*_ = 1/*l*_*ji*_ for *i*, *j* = 1,…,*n*; *i* ≠ *j*. *A* is divided into two Crisp matrices as follows:

$$A_{L}=\left[\begin{array}{cccc} 1 & l_{12} & \cdots & l_{1n}\\ l_{21} & 1 & \cdots & l_{2n}\\ \vdots & \vdots & \cdots & \vdots \\ l_{n1}\; & l_{n2} & \cdots & 1 \end{array}\right]\;\;\;\;\;\;\;\text{and}\;\;\;\;\;\;\;A_{U}=\left[\begin{array}{cccc} 1 & u_{12} & \cdots & u_{1n}\\ u_{21} & 1 & \cdots & u_{2n}\\ \vdots & \vdots & \cdots & \vdots \\ u_{n1}\; & u_{n2} & \cdots & 1 \end{array}\right],$$ (6)

where *A*_*U*_ ≤ *A* ≤ *A*_*L*_.Vector $\;W=\left([w_{1}^{L},w_{1}^{U}],\ldots ,[w_{n}^{L},\;w_{n}^{U}]\right)$ is the normalized interval weight vector, which is close to *A* in the sense that $\;a_{ij}=[l_{ij},u_{ij}]\approx [w_{i}^{L},\;w_{i}^{U}]/[w_{j}^{L},w_{j}^{U}]$ for *i*, *j* = 1,…,*n*; *i* ≠ *j*. Vector *W* is normalized [40] as follows:

$$\sum_{i}w_{i}^{U}-\underset{j}{\text{max}}(w_{j}^{U}-w_{j}^{L})\geq 1,$$ (7)

$$\sum_{i}w_{i}^{L}+\underset{j}{\text{max}}(w_{j}^{U}-w_{j}^{L})\leq 1,$$ (8)

which can be rewritten as follows:

$$w_{i}^{L}+\sum_{j=1,j\neq i}^{n}w_{j}^{U}\geq 1,$$ (9)

$$w_{i}^{U}+\sum_{j=1,j\neq i\;}^{n}w_{j}^{L}\geq 1.$$ (10)

Given that the interval comparison matrix *A* is equivalent to the weight vector *W* $\left(a_{ij}=[l_{ij},u_{ij}]\equiv [w_{i}^{L},\;w_{i}^{U}]/[w_{j}^{L},w_{j}^{U}]\right)$, *A* is written as follows:

$$A=\left[\begin{array}{cccc} 1 & \frac{[w_{1}^{L},w_{1}^{U}]}{[w_{2}^{L},w_{2}^{U}]} & \cdots & \frac{[w_{1}^{L},w_{1}^{U}]}{[w_{n}^{L},\;w_{n}^{U}]}\\ \frac{[w_{2}^{L},\;w_{2}^{U}]}{[w_{1}^{L},\;w_{1}^{U}]} & 1 & \cdots & \frac{[w_{2}^{L},\;w_{2}^{U}]}{[w_{n}^{L},\;w_{n}^{U}]}\\ \vdots & \vdots & \cdots & \vdots \\ \frac{[w_{n}^{L},\;w_{n}^{U}]}{[w_{1}^{L},\;w_{1}^{U}]} & \frac{[w_{n}^{L},\;w_{n}^{U}]}{[w_{2}^{L},\;w_{2}^{U}]} & \cdots & 1 \end{array}\right].$$ (11)

According to (Eq 4): $[w_{i}^{L},\;w_{i}^{U}]/[w_{j}^{L},\;w_{j}^{U}]=[w_{i}^{L}/w_{j}^{U},\;w_{i}^{U}/w_{j}^{L}],i\neq j$. Therefore, the interval comparison matrix *A* is rewritten as follows:

$$A=\left[\begin{array}{cccc} 1 & [\frac{w_{1}^{L}}{w_{2}^{U}},\frac{w_{1}^{U}}{w_{2}^{L}}] & \cdots & [\frac{w_{1}^{L}}{w_{n}^{U}},\frac{w_{1}^{U}}{w_{n}^{L}}]\\ {}[\frac{w_{2}^{L}}{w_{1}^{U}},\frac{w_{2}^{U}}{w_{1}^{L}}] & 1 & \cdots & [\frac{w_{2}^{L}}{w_{n}^{U}},\frac{w_{2}^{U}}{w_{n}^{L}}]\\ \vdots & \vdots & \cdots & \vdots \\ {}[\frac{w_{n}^{L}}{w_{1}^{U}},\frac{w_{n}^{U}}{w_{1}^{L}}] & [\frac{w_{n}^{L}}{w_{2}^{U}},\frac{w_{n}^{U}}{w_{2}^{L}}] & \cdots & 1 \end{array}\right].$$ (12)

*A* is divided into two Crisp matrices as follows:

$$A_{L}=\left[\begin{array}{cccc} 1 & \frac{w_{1}^{L}}{w_{2}^{U}} & \cdots & \frac{w_{1}^{L}}{w_{n}^{U}}\\ \frac{w_{2}^{L}}{w_{1}^{U}} & 1 & \cdots & \frac{w_{2}^{L}}{w_{n}^{U}}\\ \vdots & \vdots & \cdots & \vdots \\ \frac{w_{n}^{L}}{w_{1}^{U}} & \frac{w_{n}^{L}}{w_{2}^{U}} & \cdots & 1 \end{array}\right]\;\;\;\;\;\;\;\;\text{and}\;\;\;\;\;\;\;\;\;\;A_{U}=\left[\begin{array}{cccc} 1 & \frac{w_{1}^{U}}{w_{2}^{L}} & \cdots & \frac{w_{1}^{U}}{w_{n}^{L}}\\ \frac{w_{2}^{U}}{w_{1}^{L}} & 1 & \cdots & \frac{w_{2}^{U}}{w_{n}^{L}}\\ \vdots & \vdots & \cdots & \vdots \\ \frac{w_{n}^{U}}{w_{1}^{L}} & \frac{w_{n}^{U}}{w_{2}^{L}} & \cdots & 1 \end{array}\right].$$ (13)

It is easy to prove that *A*_*L*_*W*_*U*_ = *W*_*U*_ + (*n* − 1)*W*_*L*_ and *A*_*U*_*W*_*L*_ = *W*_*L*_ + (*n* − 1)*W*_*U*_, Where $\;W_{L}={\left(w_{1}^{L},\ldots ,w_{n}^{L}\right)}^{T}\;$ and $\;W_{U}={\left(w_{1}^{U},\ldots ,w_{n}^{U}\right)}^{T}$. Because the judgment of the decision maker may not be exact, the value of the deviations is defined as the following vectors:

$$E=(A_{L}-I)W_{U}-(n-1)W_{L},$$ (14)

$$\Gamma =(A_{U}-I)W_{L}-(n-1)W_{U},$$ (15)

where *E* = (*ε*_1_,…*ε*_*n*_)^*T*^, Γ = (*γ*_1_,…,*γ*_*n*_)^*T*^ and *I* is a unit matrix of order *n*. Finally, the optimization model for extracting the interval weights from an interval comparison matrix is as follows:

$$\left(M_{W}\right)\left\{ \begin{array}{l} Minimize\;\;\;J=\sum_{i=1}^{n}\left(\left|\varepsilon_{i}\right|+\left|\gamma_{i}\right|\right)\\ s.t.\\ (A_{L}-I)W_{U}-(n-1)W_{L}-E=0,\\ (A_{U}-I)W_{L}-(n-1)W_{U}-\Gamma =0,\\ w_{i}^{L}+\sum_{j=1,j\neq i}^{n}w_{j}^{U}\geq 1,\;\;\;i=1,\ldots ,n,\\ w_{i}^{U}+\sum_{j=1,j\neq i}^{n}w_{j}^{L}\geq 1,\;\;\;i=1,\ldots ,n,\\ W_{U}-W_{L}\geq 0,\\ W_{L},W_{U}\geq 0. \end{array}\right.$$

## Proposed optimization model

### Assumptions and presentation

In order to conveniently describe the model, the following notations are used:

**Parameters:**
*n*, the total number of stocks;
*k*, the desired number of stocks which can be chosen in the portfolio;
*B*, the total available budget;
*P*_*i*_, the price of stock *i*
*R*_*i*_, the return of stock *i*
*u*_*i*_, the upper bound of stock *i*
*l*_*i*_, the lower bound of stock *i*.

**Variables:**
*x*_*i*_, the integer variable that represents the number of each stock;
*y*_*i*_, the binary variable indicating whether stock *i* is included in the portfolio or not. *y*_*i*_ = 1, if stock *i* is included in the portfolio, and *y*_*i*_ = 0 otherwise;
*i* ∈ {1,2,…,*n*}.

### Model formulation

The basic model is based on the knapsack problem, which is as follows:

$$(M_{1})\{\begin{array}{ll} Maximize\;\;\;\;Z=\sum_{i=1}^{n}R_{i}x_{i} & \left(16\right)\\ s.t. & \\ \sum_{i=1}^{n}P_{i}x_{i}\leq B, & \left(17\right)\\ l_{i}y_{i}\leq x_{i}\leq u_{i}y_{i},\forall i\in \{1,2,\ldots ,n\}, & \left(18\right)\\ \sum_{i=1}^{n}y_{i}=k, & \left(19\right)\\ x_{i}\in \text{int},i\in \{1,2,\ldots ,n\}, & \left(20\right)\\ y_{i}\in \{0,1\},i\in \{1,2,\ldots ,n\}. & \left(21\right) \end{array}$$

The target function (Eq (16)) maximizes the sum of the expected returns in the stock portfolio in which the sum of the weighted prices is less than or equal to the budget (Eq (17)). (Eq 18) states that each stock should be only between its lower bound (*l*_*i*_*y*_*i*_) and its upper bound (*u*_*i*_*y*_*i*_) in case the asset is chosen, i.e. *y*_*i*_ = 1. (Eq 19) states that the portfolio should contain a certain number of stocks (*k*). All financial managers who are active on the biggest financial houses normally consider only selected number of shares, e.g. the shares of 10–12 firms, which means we need to consider cardinality constraints. Most financial managers are interested in investment in any particular company as long as they invest a minimum amount. The main reason is because they have to study all related press news of the companies on a daily basis. In fact, we needs a big investment team to follow all news and reports and literally hundreds of firms are assigned in a portfolio but when there are limited number of firms in the investment, a relatively small group could manage the fund. On the other hand, it is an easy assignment to build an example where Markowitz theorem either focuses on single investment or gives us investment on more than 10–12 firms. In many cases, the results of Markowitz theorem is impractical since one has to invest a small amount of money on one share and big portion of investment needs to be devoted on another one (see [41]). Therefore, (Eq 19) is considered for the proposed portfolio optimization problem that is the cardinality constrains.

Note that the structure of the decision variable in the knapsack-based portfolio optimization model (Eq (20)) differs from the decision variable in the mean-variance Markowitz model. In the mean-variance Markowitz model, the decision variable (the weight of each share (*w*_*i*_)) is a continuous variable. However, in the knapsack-based portfolio optimization model, the decision variable (the number of each stock (*x*_*i*_)) is an integer and/or binary variable [42,43]. In some cases, rounding the output of the Markowitz model may yield an infeasible solution or a very bad approximation to the optimal integer solution (see [31,32,35]). Therefore, this gap disrupts the optimization process and the portfolio selection model based on knapsack problem is proposed to properly allocate the number of shares to different assets. Also, the knapsack-based portfolio optimization model has an advantage over Markowitz theorem. For instance, suppose, there are three assets in a basket with the optimal weights of 0.2, 0.5 and 0.3 and we plan to invest one million dollars in these three shares whose market prices are 345,000$, 1000$ and 1400$, respectively. Consequently, the number of shares is 500, 214.285 and 0.579, respectively. As we can observe, 214.285 is not an integer number and 0.579 is less than one share. Therefore, according to Markowitz theorem; we need to purchase less than one share, which is obviously impractical. By using knapsack type optimization technique, we may be able to find the optimum asset allocation for especial cases with relatively large stock prices.

In the following, with regard to the probable risks, uncertainty conditions, and the lack of accurate information on financial markets, fuzzy and interval programming techniques have been used. In this case, instead of using the exact values, the interval values are used such that the lowest and highest expected values of the parameter are placed in the lower and upper bounds of the range, respectively. To determine the estimated values of these limits, you can use the information of past years and consult with experts. On the other hand, the risk and uncertainty conditions are not only specific to the objective function coefficients, but also the technical coefficients and the values of the right-side of the constraints may also have these conditions. Therefore, the expected return of each stock $\left(R_{i}=\left[r_{i}^{L},r_{i}^{U}\right]=\left\{R_{i}:r_{i}^{L}\leq R_{i}\leq r_{i}^{U},R_{i}\geq 0,\forall \;i\in \{1,2,\ldots ,n\}\right\}\;\right)$, the price of each stock $\left(P_{i}=\left[p_{i}^{L},p_{i}^{U}\right]\;=\left\{P_{i}:p_{i}^{L}\leq P_{i}\leq p_{i}^{U},P_{i}\geq 0,\forall \;i\in \{1,2,\ldots ,n\}\right\}\right)$, and the budget $\left(B=\left[\underline{b},\bar{b}\right]=\left\{B:\underline{b}\;\leq B\leq \bar{b},B\geq 0\right\}\;\right)$ are defined as the interval numbers. Therefore, the model is rewritten as follows:

$$\left(M_{2}\right)\left\{ \begin{array}{l} Maximize\;\;\;\;Z=\sum_{i=1}^{n}\left[r_{i}^{L},r_{i}^{U}\right]x_{i}\\ s.t.\\ \sum_{i=1}^{n}\left(\left[p_{i}^{L},p_{i}^{U}\right]x_{i}\right)\leq \left[\underline{b},\bar{b}\right],\\ l_{i}y_{i}\leq x_{i}\leq u_{i}y_{i},\forall i\in \{1,2,\ldots ,n\},\\ \sum_{i=1}^{n}y_{i}=k,\\ x_{i}\in \text{int},i\in \{1,2,\ldots ,n\},\\ y_{i}\in \{0,1\},i\in \{1,2,\ldots ,n\}. \end{array}\right.$$

As can be seen, instead of using the exact values of the coefficients, their interval values are used; For example,$\;\left[r_{i}^{L},r_{i}^{U}\right]$ in the objective function means that the target function coefficient of the *i* decision variable is possible to vary in the range $\;r_{i}^{L}\;$ to $\;r_{i}^{U}$ and to fluctuate.

In the real world, decision-making processes often have complexity and uncertainty. Sometimes it is far from the fact that judgments of decision-makers are considered accurate and definitive. For this reason, all judgments or part of them are considered as interval values or fuzzy numbers. Interval judgments can examine uncertainty in judgments without the intervention of probability distribution functions in the weight extraction models of the pairwise comparison matrix and give closer results to the decision-making in uncertainty conditions. One of the major problems in extracting weights from the interval pairwise matrix is the problem of the incompatibility of matrices containing mental judgments. Therefore, a model used to derive weights from the interval comparison matrix should be able to protect against changes to these minor incompatibilities. Two types of model responses are defined in weight extraction models: point estimates [44] and interval estimates [39]. Point estimates make decision-making processes easy, but do not reflect uncertainty in responses as an interval. Therefore, a definite answer is obtained. Interval estimates show uncertainty in decision-making processes. Also, the length of the estimated intervals can be a criterion of uncertainty. On the other hand, a stable model should be consistent with the mental incompatibilities of the decision maker, which means that it can provide responses close to the responses extracted from the compatible pairwise comparison matrix. Therefore, in order to determine the importance and priorities of each stock in the stock market, the interval weights extracting from an interval comparison matrix are added to the model (*M*_2_). Therefore, the model is written as follows:

$$\left(M_{3}\right)\left\{ \begin{array}{l} Maximize\;\;\;\;Z=\sum_{i=1}^{n}\left(\left(\left[r_{i}^{L},r_{i}^{U}\right]⊗\left[w_{i}^{L},w_{i}^{U}\right]\right)x_{i}\right)\\ s.t.\\ \sum_{i=1}^{n}\left(\left[p_{i}^{L},p_{i}^{U}\right]⊗\left[w_{i}^{L},w_{i}^{U}\right]x_{i}\right)\leq \left[\underline{b},\bar{b}\right],\\ l_{i}y_{i}\leq x_{i}\leq u_{i}y_{i},\forall i\in \{1,2,\ldots ,n\},\\ \sum_{i=1}^{n}y_{i}=k,\\ x_{i}\in \text{int},i\in \{1,2,\ldots ,n\},\\ y_{i}\in \{0,1\},i\in \{1,2,\ldots ,n\}. \end{array}\right.$$

Given these weights, the budget is also proportional to the scale. According to (Eq 2), the multiplication operator between interval values is defined as:

$$\left[r_{i}^{L},r_{i}^{U}\right]⊗\left[w_{i}^{L},w_{i}^{U}\right]={\left[\begin{array}{l} \text{min}\left(\left(r_{i}^{L}\times w_{i}^{L}\right),\left(r_{i}^{L}\times w_{i}^{U}\right),\left(r_{i}^{U}\times w_{i}^{L}\right),\left(r_{i}^{U}\times w_{i}^{U}\right)\right),\\ \text{max}\left(\left(r_{i}^{L}\times w_{i}^{L}\right),\left(r_{i}^{L}\times w_{i}^{U}\right),\left(r_{i}^{U}\times w_{i}^{L}\right),\left(r_{i}^{U}\times w_{i}^{U}\right)\right) \end{array}\right]}^{t},$$ (22)

$$\left[p_{i}^{L},p_{i}^{U}\right]⊗\left[w_{i}^{L},w_{i}^{U}\right]={\left[\begin{array}{l} \text{min}\left(\left(p_{i}^{L}\times w_{i}^{L}\right),\left(p_{i}^{L}\times w_{i}^{U}\right),\left(p_{i}^{U}\times w_{i}^{L}\right),\left(p_{i}^{U}\times w_{i}^{U}\right)\right),\\ \text{max}\left(\left(p_{i}^{L}\times w_{i}^{L}\right),\left(p_{i}^{L}\times w_{i}^{U}\right),\left(p_{i}^{U}\times w_{i}^{L}\right),\left(p_{i}^{U}\times w_{i}^{U}\right)\right) \end{array}\right]}^{t}.$$ (23)

In order to conveniently describe the model (*M*_3_), the following notations are used:

$$\text{min}\left(\left(r_{i}^{L}\times w_{i}^{L}\right),\left(r_{i}^{L}\times w_{i}^{U}\right),\left(r_{i}^{U}\times w_{i}^{L}\right),\left(r_{i}^{U}\times w_{i}^{U}\right)\right)={\underline{R}}_{i},$$ (24)

$$\text{max}\left(\left(r_{i}^{L}\times w_{i}^{L}\right),\left(r_{i}^{L}\times w_{i}^{U}\right),\left(r_{i}^{U}\times w_{i}^{L}\right),\left(r_{i}^{U}\times w_{i}^{U}\right)\right)={\bar{R}}_{i},$$ (25)

$$\text{min}\left(\left(p_{i}^{L}\times w_{i}^{L}\right),\left(p_{i}^{L}\times w_{i}^{U}\right),\left(p_{i}^{U}\times w_{i}^{L}\right),\left(p_{i}^{U}\times w_{i}^{U}\right)\right)={\underline{P}}_{i},$$ (26)

$$\text{max}\left(\left(p_{i}^{L}\times w_{i}^{L}\right),\left(p_{i}^{L}\times w_{i}^{U}\right),\left(p_{i}^{U}\times w_{i}^{L}\right),\left(p_{i}^{U}\times w_{i}^{U}\right)\right)={\bar{P}}_{i}.$$ (27)

According to Eqs (24–27), the model (*M*_3_) is rewritten as follows:

$$\left(M_{4}\right)\left\{ \begin{array}{l} Maximize\;\;\;\;Z=\sum_{i=1}^{n}\left(\left[{\underline{R}}_{i},{\bar{R}}_{i}\right]x_{i}\right)\\ s.t.\\ \sum_{i=1}^{n}\left(\left[{\underline{P}}_{i},{\bar{P}}_{i}\right]x_{i}\right)\leq \left[\underline{b},\bar{b}\right],\\ l_{i}y_{i}\leq x_{i}\leq u_{i}y_{i},\forall i\in \{1,2,\ldots ,n\},\\ \sum_{i=1}^{n}y_{i}=k,\\ x_{i}\in \text{int},i\in \{1,2,\ldots ,n\},\\ y_{i}\in \{0,1\},i\in \{1,2,\ldots ,n\}. \end{array}\right.$$

Then, the model (*M*_4_) is converted into a crisp form using the technique presented by Sengupta [45]. Therefore, a solvable parametric linear programming model is as follows:

$$\left(M_{5}\right)\left\{ \begin{array}{l} Maximize\;m(Z)=\frac{1}{2}\left(\sum_{i=1}^{n}\left(\left({\bar{R}}_{i}+{\underline{R}}_{i}\right)x_{i}\right)\right)\;\\ s.t.\\ \sum_{i=1}^{n}{\bar{P}}_{i}x_{i}\leq \bar{b},\\ \sum_{i=1}^{n}({\bar{P}}_{i}+{\underline{P}}_{i})x_{i}-(\bar{b}+\underline{b})\leq \alpha (\bar{b}-\underline{b})\;\;-\alpha \sum_{i=1}^{n}({\bar{P}}_{i}-{\underline{P}}_{i})x_{i},\\ l_{i}y_{i}\leq x_{i}\leq u_{i}y_{i},\forall \;i\in \{1,2,\ldots ,n\},\\ \sum_{i=1}^{n}y_{i}=k,\\ x_{i}\in \text{int},i\in \{1,2,\ldots ,n\},\\ y_{i}\in \{0,1\},i\in \{1,2,\ldots ,n\}, \end{array}\right.$$

where *α* (*α* ∈ [0,1]) is the data optimism threshold determined by the decision-maker. If the decision-maker is optimistic about the data, *α* is greater than 0.5 (50%) and otherwise less than 0.5. In fact, *α* is a controlling factor for data uncertainty determined by the decision-maker. Empirical studies show that it is usually better to choose *α* = 0.5 (see [46,47]).

Finally, the features and advantages of the proposed optimization model are as follows:

- The knapsack based portfolio selection model can properly allocate the number of shares to different assets.
- In the proposed optimization model, the interval uncertainty of the parameters is considered in the objective functions and constraints simultaneously. In the other words, the expected returns, prices, and budget are characterized by interval values.
- The proposed optimization model considers the risk preferences of investors. In the other words, the importance and priority of each share are considered in the proposed optimization model, which are defined as interval values.
- In the proposed optimization model, the decision-maker is able to determine the optimism threshold.
- The proposed optimization model is very suitable for the value of a particular share becomes relatively large.
- Finally, the proposed optimization model based on the knapsack problem maximizes the returns considering the risk preferences of investors, budget constraint, cardinality constraint, floor and ceiling constraints under interval uncertainty.

## Discrete firefly algorithm for portfolio selection

The complexity of the knapsack problem is NP-complete [48]. Therefore, in order to solve the proposed portfolio optimization model based on the knapsack problem with arbitrary inputs for large dimensions, we need an approximate algorithm that gives near-optimal solution. Also, a meta-heuristic method is used to solve a large-scale problem.

Many studies compared the effectiveness of the firefly algorithm with other algorithms for different types of knapsack problem (see [49–51]). They concluded that firefly algorithm and its branches are a very powerful algorithm for solving the different types of knapsack problems for both static and dynamic environments. Yang [52] introduced firefly-inspired algorithm in 2008. Then in 2010, Sayadi et al. [53] presented Discrete Firefly Algorithm (DFA). Some related researches in portfolio optimization by firefly algorithm can be found in [54,55].

In general, there are three ideal rules for the development of firefly inspired algorithms that are: 1) All fireflies are considered unisex. This means that a firefly will be absorbed into another firefly, regardless of its sex. 2) The degree of attractiveness of a firefly is proportional to its brightness. If the distance between the two fireflies increases, the brightness decreases and consequently the attractiveness decrease. If none of the fireflies are brighter than the other, the firefly will move randomly. 3) The brightness of a firefly is determined by the objective function or affected by it. Therefore:

- The attractiveness function (*β*) is defined as follows:
  $$\beta \left(r\right)=\beta_{0}\text{exp}\left(-\gamma r^{2}\right),$$ (28)
  where *r* is the distance, *β*_0_ is the attractiveness at *r* = 0 and *γ* is a fixed light absorption coefficient.
- The distance between any two fireflies *i* and *j* at locations *x*_*i*_ and *x*_*j*_ is defined as follows:
  $$r_{ij}=\left\|x_{i}-x_{j}\right\|=\sqrt{\sum_{d\in D}{\left(x_{i,d}-x_{j,d}\right)}^{2}},$$ (29)
  *D* is the dimension of the problem and *x*_*i*,*d*_ is the *d* − *th* component of the *i* − *th* firefly (*x*_*i*_).
- The movement of a firefly *i* to another more attractive (brighter) firefly *j* is defined as follows:
  $$x_{i}^{k+1}=x_{i}^{k}+\beta_{0}\text{exp}\left(-\gamma {\left(r_{ij}^{k}\right)}^{2}\right)\left(x_{j}^{k}-x_{i}^{k}\right)+\varphi \xi_{i},$$ (30)
  where *φ* is the random parameter, *ξ*_*i*_ is a vector of random numbers with Gaussian or uniform distributions in [0,1] and *k* is the iteration number.
- When a firefly *i* moves toward firefly *j*, the position of firefly *i* changes from a binary number to a real number. Therefore, the following sigmoid function is used to achieve binary position after the displacement.
  $$S\left(x_{i}^{k}\right)=\frac{1}{1+\text{exp}\left(-x_{i}^{k}\right)},$$ (31)
  where $\;S\left(x_{i}^{k}\right)\;$ is the probability of bit $\;x_{i}^{k}$ considered as 1.

The operating steps of this algorithm are as follow:

**Algorithm 1. Pseudocode of DFA.**

Suppose that *f*(*X*) is the objective function of *X* = (*x*_1_,*x*_2_,…,*x*_*d*_)^T^.

Assign value for *β*_0_, *φ*, *γ* and *MaxGeneration*.

Generate initial population of fireflies *x*_*i*_ for *i* = 1,2,…,*n*.

Determine the light intensify *I*_*i*_ at *x*_*i*_ using *f*(*x*_*i*_).

**while** (*t* < *MaxGeneration*) **do**

**for** *i* = 1: *n* all *n* fireflies **do**

**for** *i* = 1: *n* all *n* fireflies **do**

**if** (*I*_*i*_ < *I*_*j*_) **then**

Move firefly *i* towards *j*

**End if**

Vary attractiveness with distance *r* using exp(-*γr*^2^)

Discrete the position of *i* − *th* firefly (31)

Evaluate new solution (position of *i* − *th* firefly) and update light intensify *I*_*i*_

**End for**

**End for**

Rank the fireflies and find the current global best

**End while**

Show result and visualization.

## Numerical example

In this section, a numerical example is presented to express the idea of the proposed optimization model. The case study includes the shares of the Dow Jones Industrial Average (DJIA) listed in New York Stock Exchange (NYSE) and covers weekly financial time series data over a period of five years from 18/10/2013 to 18/10/2018 (Appendix A). Also, the minimum and maximum number of each share is generated randomly and interval returns are calculated from the following formula:

- The upper bound of the return = ((Minimum close price during the period- Minimum value of investment during the period+ Minimum Dividend during the period)/ Minimum value of investment during the period)*100.
- The lower bound of the return = ((Maximum value of investment during the period- Maximum close price during the period+ Maximum Dividend during the period)/ Maximum close price during the period)*100

Table 1 shows the information on the minimum and maximum number, prices and returns of each share.

**Table 1:** Research data.

	symbol	Company name	${\underline{P}}_{i}$	${\bar{P}}_{i}$	${\bar{R}}_{i}\%$	${\underline{R}}_{i}\%$	*l*_*i*_	*u*_*i*_
S_1_	BA	The Boeing Company	102.099	394.279	6.457	2.463	20	48
S_2_	GE	General Electric Company	11.210	33.000	1.771	1.094	166	180
S_3_	MMM	3M Company	120.709	259.769	2.250	0.966	15	60
S_4_	PG	The Procter & Gamble Company	65.019	94.669	5.848	2.062	34	100
S_5_	KO	The Coca-Cola Company	36.560	48.619	2.420	0.989	125	167
S_6_	AAPL	Apple Inc.	70.507	233.470	3.606	2.886	50	60
S_7_	AXP	American Express Company	50.270	111.769	4.975	1.135	33	42
S_8_	UTX	United Technologies Corporation	83.389	144.149	3.305	1.948	75	80
S_9_	CVX	Chevron Corporation	69.580	135.100	9.477	1.961	89	101
S_10_	JNJ	Johnson & Johnson	81.790	148.320	8.296	1.262	35	45
S_11_	NKE	NIKE, Inc.	34.924	86.040	2.416	0.946	111	167
S_12_	UNH	UnitedHealth Group Incorporated	66.720	272.809	1.745	1.675	12	15
S_13_	MSFT	Microsoft Corporation	33.570	116.18	4.776	1.949	45	63
S_14_	IBM	International Business Machines Corporation	116.900	199.210	4.205	2.863	21	40
S_15_	TRV	The Travelers Companies, Inc.	79.889	150.550	1.904	1.272	25	88
S_16_	MRK	Merck & Co., Inc.	44.619	72.889	2.323	2.156	71	125
S_17_	XOM	Exxon Mobil Corporation	66.550	104.720	8.608	1.646	100	120
S_18_	WMT	Wal-Mart Stores, Inc.	56.299	109.980	1.947	1.045	47	50
S_19_	GS	The Goldman Sachs Group, Inc.	138.199	275.309	2.968	1.972	55	90
S_20_	CAT	Caterpillar Inc.	56.360	173.240	6.863	2.165	23	45
S_21_	V	Visa Inc.	48.564	151.559	1.450	1.299	33	40
S_22_	CSCO	Cisco Systems, Inc.	20.250	49.470	4.969	2.363	142	176
S_23_	HD	The Home Depot, Inc.	75.480	215.429	2.531	1.916	33	66
S_24_	JPM	JPMorgan Chase & Co.	50.070	119.330	5.368	1.770	52	72
S_25_	PFE	Pfizer Inc.	27.510	45.810	2.761	2.012	142	167
S_26_	MCD	McDonald’s Corporation	87.500	178.699	3.203	0.756	25	55
S_27_	VZ	Verizon Communications Inc.	38.060	56.950	12.395	2.352	99	167
S_28_	INTC	Intel Corporation	23.400	57.599	2.789	1.434	142	167
S_29_	DIS	The Walt Disney Company	65.980	122.080	2.725	2.631	41	53
S_30_	DWDP	DowDuPont Inc.	35.110	77.080	10.680	0.676	55	120

The first step in the proposed approach is to obtain an interval comparison matrix. Therefore, a detailed questionnaire is prepared which can be found in Appendix B. Questionnaire form was sent to three experts via email. In this study, an expert is someone who has the information and operational experience on the New York Stock Exchange. In addition, the interval compare n matrix of DJIA can be found in Appendix C.

The second step in the proposed approach is to extract the interval weights from an interval comparison matrix. For this purpose, interval weights are obtained using the Wang’s approach [39] and are reported in Table 2.

**Table 2:** Extracting interval weights from the interval comparison matrix.

Weight of shares		
****$W_{S_{1}}=\left[0.074,0.095\right]$****	$W_{S_{2}}=\left[0.005,0.006\right]$	$W_{S_{3}}=\left[0.055,0.065\right]$
****$W_{S_{4}}=\left[0.020,0.024\right]$****	$W_{S_{5}}=\left[0.006,0.008\right]$	$W_{S_{6}}=\left[0.042,0.054\right]$
****$W_{S_{7}}=\left[0.024,0.030\right]$****	$W_{S_{8}}=\left[0.028,0.032\right]$	$W_{S_{9}}=\left[0.028,0.032\right]$
****$W_{S_{10}}=\left[0.032,0.038\right]$****	$W_{S_{11}}=\left[0.006,0.009\right]$	$W_{S_{12}}=\left[0.068,0.079\right]$
****$W_{S_{13}}=\left[0.016,0.022\right]$****	$W_{S_{14}}=\left[0.037,0.047\right]$	$W_{S_{15}}=\left[0.032,0.039\right]$
****$W_{S_{16}}=\left[0.008,0.014\right]$****	$W_{S_{17}}=\left[0.015,0.020\right]$	$W_{S_{18}}=\left[0.020,0.021\right]$
****$W_{S_{19}}=\left[0.062,0.075\right]$****	$W_{S_{20}}=\left[0.033,0.042\right]$	$W_{S_{21}}=\left[0.025,0.030\right]$
****$W_{S_{22}}=\left[0.006,0.007\right]$****	$W_{S_{23}}=\left[0.054,0.059\right]$	$W_{S_{24}}=\left[0.014,0.019\right]$
****$W_{S_{25}}=\left[0.006,0.007\right]$****	$W_{S_{26}}=\left[0.052,0.059\right]$	$W_{S_{27}}=\left[0.006,0.010\right]$
****$W_{S_{28}}=\left[0.006,0.007\right]$****	$W_{S_{29}}=\left[0.019,0.024\right]$	$W_{S_{30}}=\left[0.013,0.018\right]$

In the following, the optimization model with the interval weights, the interval prices and the interval expected returns is solved in GAMS software. Table 3 shows the results of the exact solution of the proposed optimization model based on the different values of the uncertain parameter (*α* = {0,0.1.0.3,0.5,0.7,1}) in small dimensions.

**Table 3:** Exact solution.

GAMS solution						
	*α* = 0	*α* = 0.1	*α* = 0.3	*α* = 0.5	*α* = 0.7	*α* = 1
K = 6	E(R) = 49.234% X_4_ = 36; X_9_ = 97; X_16_ = 72; X_17_ = 100; X_27_ = 100; X_30_ = 100.	E(R) = 50.679% X_4_ = 46; X_9_ = 100; X_16_ = 72; X_17_ = 100; X_27_ = 100; X_30_ = 100.	E(R) = 53.378% X_4_ = 76; X_9_ = 100; X_16_ = 71; X_17_ = 100; X_27_ = 100; X_30_ = 100.	E(R) = 55.782% X_4_ = 100; X_9_ = 100; X_16_ = 80; X_17_ = 100; X_27_ = 100; X_30_ = 100.	E(R) = 57.744% X_4_ = 75; X_9_ = 100; X_10_ = 35; X_17_ = 100; X_27_ = 100; X_30_ = 100.	E(R) = 60.813% X_4_ = 99; X_9_ = 100; X_10_ = 40; X_17_ = 100; X_27_ = 100; X_30_ = 100
K = 7	E(R) = 46.974% X_4_ = 34; X_9_ = 89; X_13_ = 45; X_16_ = 71; X_17_ = 100; X_27_ = 99; X_30_ = 64.	E(R) = 49.254% X_4_ = 34; X_9_ = 89; X_13_ = 45; X_16_ = 71; X_17_ = 100; X_27_ = 100; X_30_ = 86.	E(R) = 52.455% X_4_ = 36; X_9_ = 98; X_13_ = 45; X_16_ = 71; X_17_ = 100; X_27_ = 100; X_30_ = 100.	E(R) = 54.981% X_4_ = 58; X_9_ = 100; X_13_ = 45; X_16_ = 71; X_17_ = 100; X_27_ = 100; X_30_ = 100.	E(R) = 57.038% X_4_ = 80; X_9_ = 100; X_16_ = 71; X_17_ = 100; X_24_ = 52; X_27_ = 100;. X_30_ = 100.	E(R) = 59.925% X_4_ = 79; X_9_ = 100; X_10_ = 35; X_16_ = 73; X_17_ = 100; X_27_ = 100; X_30_ = 100.
K = 8	E(R) = 45.625% X_4_ = 86; X_7_ = 33; X_13_ = 45; X_16_ = 71; X_17_ = 100; X_24_ = 52; X_27_ = 100; X_30_ = 100.	E(R) = 46.860% X_4_ = 98; X_7_ = 33; X_13_ = 46; X_16_ = 73; X_17_ = 100; X_24_ = 52; X_27_ = 100; X_30_ = 100.	E(R) = 49.484% X_4_ = 34; X_7_ = 33; X_9_ = 89; X_13_ = 45; X_16_ = 71; X_17_ = 100; X_27_ = 99; X_30_ = 60.	E(R) = 53.303% X_4_ = 34; X_7_ = 33; X_9_ = 89; X_13_ = 45; X_16_ = 71; X_17_ = 100; X_27_ = 99; X_30_ = 98.	E(R) = 55.749% X_4_ = 34; X_9_ = 99; X_13_ = 45; X_16_ = 71; X_17_ = 100; X_24_ = 52; X_27_ = 100;. X_30_ = 100	E(R) = 58.682% X_4_ = 68; X_7_ = 33; X_9_ = 99; X_16_ = 71; X_17_ = 100; X_24_ = 52; X_27_ = 100; X_30_ = 100.
K = 9	E(R) = 43.762% X_4_ = 34; X_7_ = 33; X_13_ = 45; X_16_ = 71; X_17_ = 100; X_20_ = 23; X_24_ = 52; X_27_ = 100;. X_30_ = 87.	E(R) = 45.431% X_4_ = 38; X_7_ = 33; X_13_ = 45; X_16_ = 71; X_17_ = 100; X_20_ = 20; X_24_ = 52; X_27_ = 100; X_30_ = 100.	E(R) = 47.906% X_4_ = 42; X_7_ = 33; X_10_ = 35; X_13_ = 45; X_16_ = 72; X_17_ = 100; X_24_ = 52; X_27_ = 100; X_30_ = 100.	E(R) = 50.061% X_4_ = 66; X_7_ = 33; X_10_ = 35; X_13_ = 45; X_16_ = 71; X_17_ = 100; X_24_ = 52; X_27_ = 100; X_30_ = 100.	E(R) = 52.480% X_4_ = 34; X_7_ = 33; X_9_ = 89; X_13_ = 45; X_16_ = 71; X_17_ = 100; X_24_ = 52; X_27_ = 99; X_30_ = 57.	E(R) = 57.077% X_4_ = 36; X_7_ = 33; X_9_ = 89; X_13_ = 45; X_16_ = 72; X_17_ = 100; X_24_ = 52; X_27_ = 100; X_30_ = 100.
K = 10	E(R) = 36.058% X_4_ = 34; X_7_ = 33; X_13_ = 45; X_16_ = 71; X_18_ = 47; X_20_ = 23; X_24_ = 52; X_27_ = 99; X_29_ = 41; X_30_ = 71.	E(R) = 40.326% X_4_ = 34; X_7_ = 33; X_13_ = 45; X_16_ = 71; X_17_ = 100; X_18_ = 47; X_24_ = 52; X_27_ = 100; X_29_ = 41; X_30_ = 56.	E(R) = 44.145% X_4_ = 34; X_7_ = 33; X_13_ = 45; X_16_ = 71; X_17_ = 100; X_18_ = 47; X_24_ = 52; X_27_ = 100; X_29_ = 41; X_30_ = 94.	E(R) = 47.032% X_4_ = 34; X_7_ = 33; X_13_ = 45; X_16_ = 71; X_17_ = 100; X_20_ = 23; X_24_ = 52; X_27_ = 100; X_29_ = 41; X_30_ = 96.	E(R) = 49.702% X_4_ = 36; X_7_ = 33; X_10_ = 35; X_13_ = 45; X_16_ = 71; X_17_ = 100; X_24_ = 52; X_27_ = 100; X_29_ = 41; X_30_ = 100.	E(R) = 52.426% X_4_ = 66; X_7_ = 33; X_10_ = 35; X_13_ = 45; X_16_ = 71; X_17_ = 100; X_24_ = 52; X_27_ = 100; X_29_ = 41; X_30_ = 100.

As shown in Table 3, the solution of the model (*M*_5_) is not unique. This means that different values of *α* yield various solutions. When *α* is equal to zero, the expected return of the portfolio is minimum. When the level of data satisfaction (*α*) increases, the expected return of the portfolio will also increase. When *α* is equal to one, the expected return of the portfolio is maximum. For instance, the expected return of the portfolio with cardinality *k = 6* lies in [0.49234 0.60813] based on different values of *α* (*α* ∈ [0,1]).

The proposed portfolio selection model based on the knapsack problem is a mixed-integer optimization problem. Therefore, the exact methods such as the implementation of GAMS software package does not yield appropriate answers for large-size problems. In order to use the proposed model in the large scale, it is necessary to use the meta-heuristic algorithms. Therefore, Discrete Firefly Algorithm (DFA) is used.

To select appropriate parameters, the Taguchi method is used for five parameters of DFA (Number of iterations, Number of fireflies, Randomization parameter, Absorption coefficient, Attractiveness). Finally, the selected parameters of this algorithm are attractiveness (*β*_0_ = 1), randomization parameter (*φ* = 0.2), absorption coefficient (*γ* = 1), number of iterations (*MaxGeneration* = 500), and number of fireflies (*Population* = 30).

The results of discrete firefly algorithm in small dimensions are reported in Table 4. Then, the results of the exact method are compared with the results of DFA and are reported in Table 5:

**Table 4:** DFA solution.

t	Init. assign. no.	DFAsolution					
K = 6		***α* = 0**	***α* = 0.1**	***α* = 0.3**	***α* = 0.5**	***α* = 0.7**	***α* = 1**
	1	50.971%	52.527%	53.048%	55.618%	55.289%	57.867%
	2	50.939%	50.895%	52.507%	54.336%	55.936%	57.923%
	3	49.451%	50.331%	52.352%	55.624%	56.744%	57.788%
	4	49.828%	52.194%	52.804%	55.419%	56.680%	58.604%
	5	50.987%	51.459%	53.029%	54.610%	55.638%	58.441%
Mean		50.435%	51.481%	52.748%	55.121%	56.057%	58.125%
SE Mean		0.003	0.004	0.001	0.002	0.002	0.001
S.D.		0.007	0.009	0.003	0.006	0.006	0.003
K = 7		***α* = 0**	***α* = 0.1**	***α* = 0.3**	***α* = 0.5**	***α* = 0.7**	***α* = 1**
	1	53.469%	54.087%	54.431%	56.192%	58.116%	57.534%
	2	52.830%	54.220%	55.039%	57.179%	58.503%	59.046%
	3	53.923%	53.005%	54.424%	57.781%	56.723%	58.635%
	4	53.580%	53.969%	53.859%	56.617%	57.617%	59.180%
	5	53.282%	53.656%	55.539%	54.825%	57.236%	59.041%
Mean		53.417%	53.787%	54.658%	56.519%	57.639%	58.687%
SE Mean		0.001	0.002	0.002	0.005	0.003	0.003
S.D.		0.004	0.004	0.006	0.011	0.007	0.006
K = 8		***α* = 0**	***α* = 0.1**	***α* = 0.3**	***α* = 0.5**	***α* = 0.7**	***α* = 1**
	1	53.160%	53.692%	54.438%	57.332%	59.896%	61.281%
	2	51.837%	56.064%	57.462%	57.531%	59.483%	62.910%
	3	52.511%	52.538%	55.797%	58.439%	59.061%	62.089%
	4	53.437%	53.928%	57.652%	58.694%	59.821%	60.880%
	5	53.823%	54.777%	54.765%	58.648%	58.930%	60.379%
Mean		52.954%	54.200%	56.023%	58.129%	59.438%	61.508%
SE Mean		0.003	0.005	0.006	0.002	0.001	0.004
S.D.		0.007	0.013	0.014	0.006	0.004	0.010
K = 9		***α* = 0**	***α* = 0.1**	***α* = 0.3**	***α* = 0.5**	***α* = 0.7**	***α* = 1**
	1	53.268%	56.171%	56.016%	57.217%	60.927%	61.928%
	2	54.554%	56.412%	58.045%	59.243%	59.971%	61.676%
	3	52.942%	54.454%	58.006%	57.593%	58.478%	62.929%
	4	52.866%	55.073%	57.444%	58.143%	59.191%	62.821%
	5	52.616%	54.091%	56.257%	58.585%	60.941%	62.020%
Mean		53.249%	55.240%	57.154%	58.156%	59.902%	62.275%
SE Mean		0.003	0.004	0.004	0.003	0.004	0.002
S.D.		0.007	0.010	0.009	0.008	0.010	0.005
K = 10		***α* = 0**	***α* = 0.1**	***α* = 0.3**	***α* = 0.5**	***α* = 0.7**	***α* = 1**
	1	54.988%	55.175%	56.752%	59.166%	60.544%	63.510%
	2	52.911%	52.341%	56.883%	58.985%	61.030%	62.007%
	3	53.477%	53.014%	57.139%	58.885%	59.531%	63.767%
	4	53.942%	54.696%	57.481%	59.369%	60.470%	63.151%
	5	51.117%	55.491%	55.189%	58.697%	61.679%	63.087%
Mean		53.287%	54.143%	56.689%	59.020%	60.651%	63.104%
SE Mean		0.006	0.006	0.003	0.001	0.003	0.003
S.D.		0.014	0.013	0.008	0.002	0.007	0.006

**Table 5:** Comparison of the exact solution with DFA solution.

		Mean solution of DFA	GAMS solution	S.D.	SE Mean	P-Value*
K = 6	***α* = 0**	50.435%	49.234%	0.008	0.006	0.008
	***α* = 0.1**	51.481%	50.679%	0.005	0.004	0.005
	***α* = 0.3**	52.748%	53.378%	0.004	0.003	0.004
	***α* = 0.5**	55.121%	55.782%	0.004	0.003	0.004
	***α* = 0.7**	56.057%	57.744%	0.011	0.008	0.010
	***α* = 1**	58.125%	60.813%	0.019	0.013	0.015
K = 7	***α* = 0**	53.417%	46.974%	0.045	0.032	0.042
	***α* = 0.1**	53.787%	49.254%	0.032	0.022	0.029
	***α* = 0.3**	54.658%	52.455%	0.015	0.011	0.013
	***α* = 0.5**	56.519%	54.811%	0.012	0.008	0.010
	***α* = 0.7**	57.639%	57.038%	0.004	0.003	0.003
	***α* = 1**	58.687%	59.925%	0.008	0.006	0.007
K = 8	***α* = 0**	52.954%	45.652%	0.051	0.036	0.048
	***α* = 0.1**	54.200%	46.860%	0.051	0.036	0.047
	***α* = 0.3**	56.023%	49.489%	0.046	0.032	0.040
	***α* = 0.5**	58.129%	53.303%	0.034	0.024	0.028
	***α* = 0.7**	59.438%	55.749%	0.026	0.018	0.021
	***α* = 1**	61.508%	58.682%	0.020	0.014	0.015
K = 9	***α* = 0**	53.249%	43.762%	0.067	0.047	0.063
	***α* = 0.1**	55.240%	45.431%	0.069	0.049	0.063
	***α* = 0.3**	57.154%	47.906%	0.065	0.046	0.057
	***α* = 0.5**	58.156%	50.061%	0.057	0.040	0.048
	***α* = 0.7**	59.902%	52.480%	0.052	0.037	0.043
	***α* = 1**	62.275%	57.077%	0.036	0.026	0.028
K = 10	***α* = 0**	53.287%	36.058%	0.121	0.086	0.124
	***α* = 0.1**	54.143%	40.326%	0.097	0.069	0.094
	***α* = 0.3**	56.689%	44.145%	0.088	0.062	0.080
	***α* = 0.5**	59.020%	47.032%	0.084	0.059	0.073
	***α* = 0.7**	60.651%	49.702%	0.077	0.054	0.064
	***α* = 1**	63.104%	52.426%	0.075	0.053	0.060

* denotes rejection of the hypothesis at the 0.01 level.

As shown in Table 5, Standard Deviations (SD) and Standard Errors of the Mean (SE Mean) between GAMS solutions and DFA solutions lie in [0.004 0.121] and [0.003 0.086], respectively. These results are very reasonable and show that DFA solutions are very close to the exact solutions in small dimensions. Also, as shown in S1 Fig, as the data optimism threshold increases, the results of the two approaches converge more into each other. Therefore, for *α* greater than 0.5 (*α*≥0.5), the results of the DFA are more valid.

Finally, these results indicate the validity of the used discrete firefly algorithm for the proposed portfolio selection model. Therefore, this algorithm can be used to solve the proposed optimization model in larger dimensions as well. Therefore, the sensitivity analysis and the set of answers in small and large dimensions are reported in Table 6:

**Table 6:** Set of answers in small and large dimension.

	D	DFA Solution						GAMS Solution					
		*α* = 0	*α* = 0.1	*α* = 0.3	*α* = 0.5	*α* = 0.7	*α* = 1	*α* = 0	*α* = 0.1	*α* = 0.3	*α* = 0.5	*α* = 0.7	*α* = 1
Low -D	K = 6	50.435%	51.481%	52.748%	55.121%	56.057%	58.125%	49.234%	50.679%	53.378%	55.782%	57.744%	60.813%
	K = 7	53.417%	53.787%	54.658%	56.519%	57.639%	58.687%	46.974%	49.254%	52.455%	54.811%	57.038%	59.925%
	K = 8	52.954%	54.200%	56.023%	58.129%	59.438%	61.508%	45.652%	46.860%	49.489%	53.303%	55.749%	58.682%
	K = 9	53.249%	55.240%	57.154%	58.156%	59.902%	62.275%	36.058%	40.326%	44.145%	47.032%	49.702%	52.426%
	K = 10	53.287%	54.143%	56.689%	59.020%	60.651%	63.104%	36.058%	40.326%	44.145%	47.032%	49.702%	52.426%
High- D	K = 11	52.664%	55.066%	56.087%	59.616%	62.251%	63.694%	-	-	-	-	-	-
	K = 12	50.432%	52.244%	55.373%	58.155%	60.899%	63.898%	-	-	-	-	-	-
	K = 13	48.351%	49.467%	52.614%	55.060%	57.667%	61.688%	-	-	-	-	-	-
	K = 14	47.935%	48.951%	49.584%	52.767%	55.157%	57.829%	-	-	-	-	-	-
	K = 15	44.075%	45.247%	47.652%	49.948%	52.576%	55.531%	-	-	-	-	-	-

According to Table 6, the proposed discrete firefly algorithm is appropriate for solving the proposed portfolio selection model based on the knapsack problem with arbitrary inputs (different cardinalities) in large dimensions. Therefore, in the large-scale optimizations with a relatively large portfolio, the proposed discrete firefly algorithm is used.

## Conclusions

In this paper, we have presented a portfolio selection model based on the knapsack problem under interval uncertainty, in which the expected returns, prices, and budget are characterized by interval values. We have also considered the importance and the priority of each share in the portfolio optimization model by extracting the interval weights from an interval comparison matrix. Finally, we have converted the proposed model into a crisp form and a solvable parametric linear programming model using the technique presented by Sengupta [45] in which the decision maker is able to determine the optimism threshold.

The proposed optimization model has maximized the returns of the investment by taking into account the risk preferences of investors and budget, cardinality, floor and ceiling constraints under interval uncertainty of the parameters in the objective functions and constraints, simultaneously. The proposed model has the ability to determine the optimal portfolio of assets. In fact, the main contribution of this work is to present a portfolio selection model based on the knapsack problem, which eliminates the mentioned gaps in the Markowitz theorem. The portfolio selection model based on knapsack problem is proposed to properly allocate the number of shares to different assets. In addition, by using knapsack type optimization technique, we may be able to find the optimum asset allocation for especial cases with relatively large stock prices.

A discrete firefly algorithm has also been designed to handle the complexity of the problem. A numerical example of the US stock exchange has been given to illustrate the application of the proposed model and demonstrated the effectiveness of the designed algorithm for solving the proposed model. Numerical results have shown that the proposed optimization model and the implementation of discrete firefly algorithm may yield promising results.

Finally, the inclusion of other constraints in the proposed model such as class constraint and chance constraints and the inclusion of risk measurement factors such as variance and Value-at-Risk (VaR) will be an interesting extension of the proposed model and is regarded as the future scope of the study.

## Supporting information

S1 Fig Fig A. **Comparison of the exact solution with DFA solution.** Fig B. **Comparison of the exact solution with DFA solution.** Fig C. **Comparison of the exact solution with DFA solution.** Fig D. **Comparison of the exact solution with DFA solution.** Fig E. **Comparison of the exact solution with DFA solution.**
(TIFF)

S1 Table Research data. (PDF)

S2 Table Extracting interval weights from the interval comparison matrix. (PDF)

S3 Table Exact solution. (PDF)

S4 Table DFA solution. (PDF)

S5 Table Comparison of the exact solution with DFA solution. (PDF)

S6 Table Set of answers in small and large dimension. (PDF)

S1 Algorithm Pseudocode of DFA. (PDF)

S1 Appendix Appendix A. Data related to the article.
(XLSX)

S2 Appendix Appendix B. Survey form.
(PDF)

S3 Appendix Appendix C. Interval comparison matrix.
(PDF)
