# Supplementary figures and images for: A portfolio selection model based on the knapsack problem under uncertainty
Source: PLoS One. 2019 May 1;14(5):e0213652. doi: 10.1371/journal.pone.0213652 (PMC6493714; doi:10.1371/journal.pone.0213652)

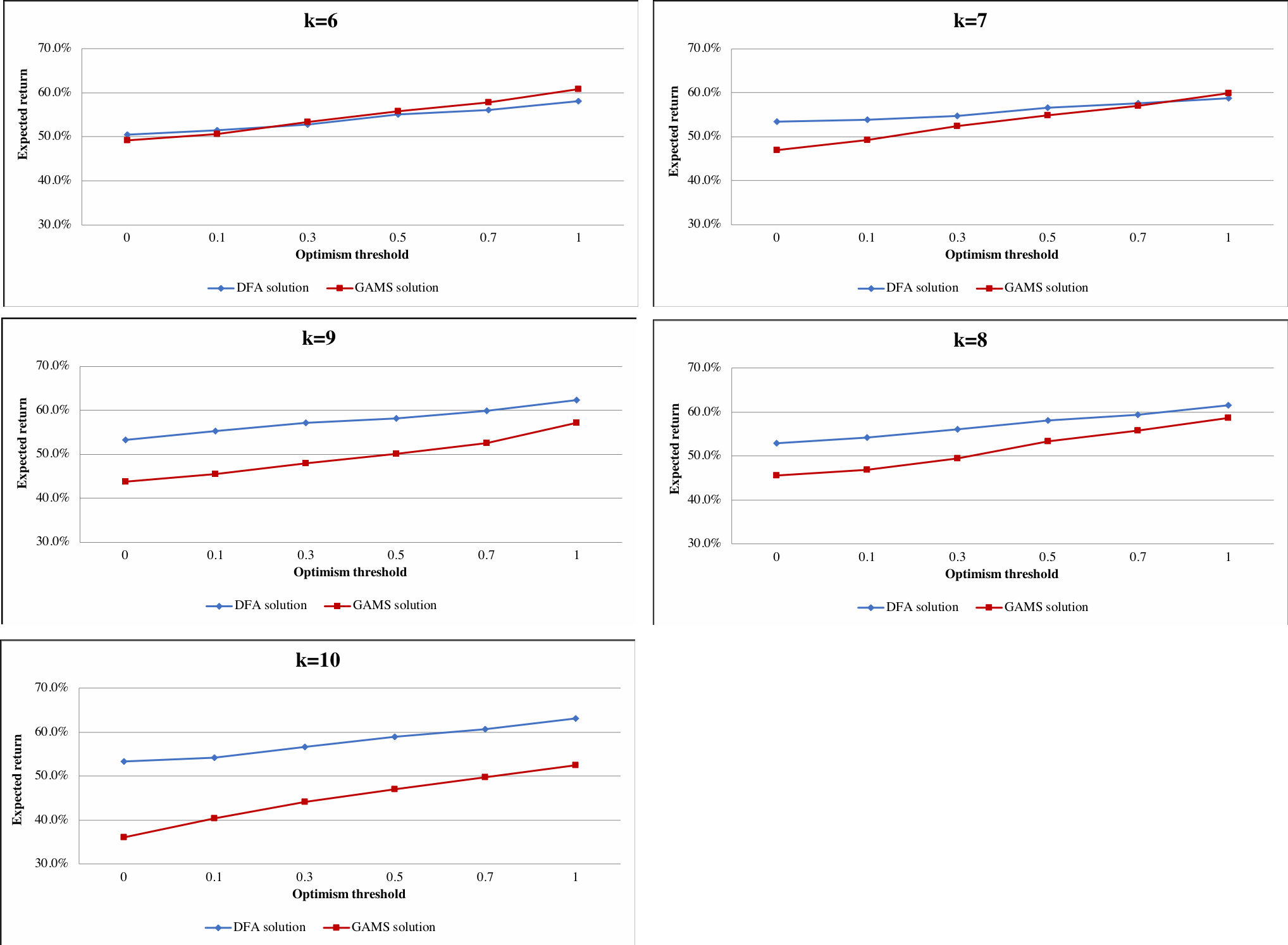

Supplement: S1 Fig — Fig A. Comparison of the exact solution with DFA solution. Fig B. Comparison of the exact solution with DFA solution. Fig C. Comparison of the exact solution with DFA solution. Fig D. Comparison of the exact solution with DFA solution. Fig E. Comparison of the exact solution with DFA solution. (TIFF) [file pone.0213652.s001.tiff]
