# Supplementary material for: A portfolio selection model based on the knapsack problem under uncertainty
Source: PLoS One. 2019 May 1;14(5):e0213652. doi: 10.1371/journal.pone.0213652 (PMC6493714; doi:10.1371/journal.pone.0213652)
Supplement: S1 Table — (PDF) [file pone.0213652.s002.pdf]

	symbol	Company name	$\underline{P}_i$	$\overline{P}_i$	$\overline{R}_i\%$	$\underline{R}_i\%$	l_i	u_i
S_1	BA	The Boeing Company	102.099	394.279	6.457	2.463	20	48
S_2	GE	General Electric Company	11.210	33.000	1.771	1.094	166	180
S_3	MMM	3M Company	120.709	259.769	2.250	0.966	15	60
S_4	PG	The Procter & Gamble Company	65.019	94.669	5.848	2.062	34	100
S_5	KO	The Coca-Cola Company	36.560	48.619	2.420	0.989	125	167
S_6	AAPL	Apple Inc.	70.507	233.470	3.606	2.886	50	60
S_7	AXP	American Express Company	50.270	111.769	4.975	1.135	33	42
S_8	UTX	United Technologies Corporation	83.389	144.149	3.305	1.948	75	80
S_9	CVX	Chevron Corporation	69.580	135.100	9.477	1.961	89	101
S_{10}	JNJ	Johnson & Johnson	81.790	148.320	8.296	1.262	35	45
S_{11}	NKE	NIKE, Inc.	34.924	86.040	2.416	0.946	111	167
S_{12}	UNH	UnitedHealth Group Incorporated	66.720	272.809	1.745	1.675	12	15
S_{13}	MSFT	Microsoft Corporation	33.570	116.18	4.776	1.949	45	63
S_{14}	IBM	International Business Machines Corporation	116.900	199.210	4.205	2.863	21	40
S_{15}	TRV	The Travelers Companies, Inc.	79.889	150.550	1.904	1.272	25	88
S_{16}	MRK	Merck & Co., Inc.	44.619	72.889	2.323	2.156	71	125
S_{17}	XOM	Exxon Mobil Corporation	66.550	104.720	8.608	1.646	100	120
S_{18}	WMT	Wal-Mart Stores, Inc.	56.299	109.980	1.947	1.045	47	50
S_{19}	GS	The Goldman Sachs Group, Inc.	138.199	275.309	2.968	1.972	55	90
S_{20}	CAT	Caterpillar Inc.	56.360	173.240	6.863	2.165	23	45
S_{21}	V	Visa Inc.	48.564	151.559	1.450	1.299	33	40
S_{22}	CSCO	Cisco Systems, Inc.	20.250	49.470	4.969	2.363	142	176
S_{23}	HD	The Home Depot, Inc.	75.480	215.429	2.531	1.916	33	66
S_{24}	JPM	JPMorgan Chase & Co.	50.070	119.330	5.368	1.770	52	72
S_{25}	PFE	Pfizer Inc.	27.510	45.810	2.761	2.012	142	167
S_{26}	MCD	McDonald's Corporation	87.500	178.699	3.203	0.756	25	55
S_{27}	VZ	Verizon Communications Inc.	38.060	56.950	12.395	2.352	99	167
S_{28}	INTC	Intel Corporation	23.400	57.599	2.789	1.434	142	167
S_{29}	DIS	The Walt Disney Company	65.980	122.080	2.725	2.631	41	53
S_{30}	DWDP	DowDuPont Inc.	35.110	77.080	10.680	0.676	55	120
