# Supplementary material for: A portfolio selection model based on the knapsack problem under uncertainty
Source: PLoS One. 2019 May 1;14(5):e0213652. doi: 10.1371/journal.pone.0213652 (PMC6493714; doi:10.1371/journal.pone.0213652)
Supplement: S2 Table — (PDF) [file pone.0213652.s003.pdf]

Weight of shares		
$W_{S_1} = [0.074, 0.095]$	$W_{S_2} = [0.005, 0.006]$	$W_{S_3} = [0.055, 0.065]$
$W_{S_4} = [0.020, 0.024]$	$W_{S_5} = [0.006, 0.008]$	$W_{S_6} = [0.042, 0.054]$
$W_{S_7} = [0.024, 0.030]$	$W_{S_8} = [0.028, 0.032]$	$W_{S_9} = [0.028, 0.032]$
$W_{S_{10}} = [0.032, 0.038]$	$W_{S_{11}} = [0.006, 0.009]$	$W_{S_{12}} = [0.068, 0.079]$
$W_{S_{13}} = [0.016, 0.022]$	$W_{S_{14}} = [0.037, 0.047]$	$W_{S_{15}} = [0.032, 0.039]$
$W_{S_{16}} = [0.008, 0.014]$	$W_{S_{17}} = [0.015, 0.020]$	$W_{S_{18}} = [0.020, 0.021]$
$W_{S_{19}} = [0.062, 0.075]$	$W_{S_{20}} = [0.033, 0.042]$	$W_{S_{21}} = [0.025, 0.030]$
$W_{S_{22}} = [0.006, 0.007]$	$W_{S_{23}} = [0.054, 0.059]$	$W_{S_{24}} = [0.014, 0.019]$
$W_{S_{25}} = [0.006, 0.007]$	$W_{S_{26}} = [0.052, 0.059]$	$W_{S_{27}} = [0.006, 0.010]$
$W_{S_{28}} = [0.006, 0.007]$	$W_{S_{29}} = [0.019, 0.024]$	$W_{S_{30}} = [0.013, 0.018]$
