# Supplementary material for: A portfolio selection model based on the knapsack problem under uncertainty
Source: PLoS One. 2019 May 1;14(5):e0213652. doi: 10.1371/journal.pone.0213652 (PMC6493714; doi:10.1371/journal.pone.0213652)
Supplement: S3 Table — (PDF) [file pone.0213652.s004.pdf]

GAMS solution						
	$\alpha = 0$	$\alpha = 0.1$	$\alpha = 0.3$	$\alpha = 0.5$	$\alpha = 0.7$	$\alpha = 1$
K=6	E(R)= 49.234% X_4=36; X_9=97; X_{16}=72; X_{17}=100; X_{27}=100; X_{30}=100.	E(R)= 50.679% X_4=46; X_9=100; X_{16}=72; X_{17}=100; X_{27}=100; X_{30}=100.	E(R)= 53.378% X_4=76; X_9=100; X_{16}=71; X_{17}=100; X_{27}=100; X_{30}=100.	E(R)= 55.782% X_4=100; X_9=100; X_{16}=80; X_{17}=100; X_{27}=100; X_{30}=100.	E(R)= 57.744% X_4=75; X_9=100; X_{10}=35; X_{17}=100; X_{27}=100; X_{30}=100.	E(R)= 60.813% X_4=99; X_9=100; X_{10}=40; X_{17}=100; X_{27}=100; X_{30}=100
K=7	E(R)= 46.974% X_4=34; X_9=89; X_{13}=45; X_{16}=71; X_{17}=100; X_{27}=99; X_{30}=64.	E(R)= 49.254% X_4=34; X_9=89; X_{13}=45; X_{16}=71; X_{17}=100; X_{27}=100; X_{30}=86.	E(R)= 52.455% X_4=36; X_9=98; X_{13}=45; X_{16}=71; X_{17}=100; X_{27}=100; X_{30}=100.	E(R)= 54.981% X_4=58; X_9=100; X_{13}=45; X_{16}=71; X_{17}=100; X_{27}=100; X_{30}=100.	E(R)= 57.038% X_4=80; X_9=100; X_{16}=71; X_{17}=100; X_{24}=52; X_{27}=100;. X_{30}=100.	E(R)= 59.925% X_4=79; X_9=100; X_{10}=35; X_{16}=73; X_{17}=100; X_{27}=100; X_{30}=100.
K=8	E(R)=45.625% X_4=86; X_7=33; X_{13}=45; X_{16}=71; X_{17}=100; X_{24}=52; X_{27}=100; X_{30}=100.	E(R)=46.860% X_4=98; X_7=33; X_{13}=46; X_{16}=73; X_{17}=100; X_{24}=52; X_{27}=100; X_{30}=100.	E(R)=49.484% X_4=34; X_7=33; X_9=89; X_{13}=45; X_{16}=71; X_{17}=100; X_{27}=99; X_{30}=60.	E(R)=53.303% X_4=34; X_7=33; X_9=89; X_{13}=45; X_{16}=71; X_{17}=100; X_{27}=99; X_{30}=98.	E(R)=55.749% X_4=34; X_9=99; X_{13}=45; X_{16}=71; X_{17}=100; X_{24}=52; X_{27}=100;. X_{30}=100	E(R)=58.682% X_4=68; X_7=33; X_9=99; X_{16}=71; X_{17}=100; X_{24}=52; X_{27}=100; X_{30}=100.
K=9	E(R)=43.762% X_4=34; X_7=33; X_{13}=45; X_{16}=71; X_{17}=100; X_{20}=23; X_{24}=52; X_{27}=100;. X_{30}=87.	E(R)=45.431% X_4=38; X_7=33; X_{13}=45; X_{16}=71; X_{17}=100; X_{20}=20; X_{24}=52; X_{27}=100; X_{30}=100.	E(R)=47.906% X_4=42; X_7=33; X_{10}=35; X_{13}=45; X_{16}=72; X_{17}=100; X_{24}=52; X_{27}=100; X_{30}=100.	E(R)= 50.061% X_4=66; X_7=33; X_{10}=35; X_{13}=45; X_{16}=71; X_{17}=100; X_{24}=52; X_{27}=100; X_{30}=100.	E(R)=52.480% X_4=34; X_7=33; X_9=89; X_{13}=45; X_{16}=71; X_{17}=100; X_{24}=52; X_{27}=99; X_{30}=57.	E(R)=57.077% X_4=36; X_7=33; X_9=89; X_{13}=45; X_{16}=72; X_{17}=100; X_{24}=52; X_{27}=100; X_{30}=100.
K=10	E(R)=36.058% X_4=34; X_7=33; X_{13}=45; X_{16}=71; X_{18}=47; X_{20}=23; X_{24}=52; X_{27}=99; X_{29}=41; X_{30}=71.	E(R)=40.326% X_4=34; X_7=33; X_{13}=45; X_{16}=71; X_{17}=100; X_{18}=47; X_{24}=52; X_{27}=100; X_{29}=41; X_{30}=56.	E(R)=44.145% X_4=34; X_7=33; X_{13}=45; X_{16}=71; X_{17}=100; X_{18}=47; X_{24}=52; X_{27}=100; X_{29}=41; X_{30}=94.	E(R)=47.032% X_4=34; X_7=33; X_{13}=45; X_{16}=71; X_{17}=100; X_{20}=23; X_{24}=52; X_{27}=100; X_{29}=41; X_{30}=96.	E(R)=49.702% X_4=36; X_7=33; X_{10}=35; X_{13}=45; X_{16}=71; X_{17}=100; X_{24}=52; X_{27}=100; X_{29}=41; X_{30}=100.	E(R)=52.426% X_4=66; X_7=33; X_{10}=35; X_{13}=45; X_{16}=71; X_{17}=100; X_{24}=52; X_{27}=100; X_{29}=41; X_{30}=100.
