# Supplementary material for: A portfolio selection model based on the knapsack problem under uncertainty
Source: PLoS One. 2019 May 1;14(5):e0213652. doi: 10.1371/journal.pone.0213652 (PMC6493714; doi:10.1371/journal.pone.0213652)
Supplement: S4 Table — (PDF) [file pone.0213652.s005.pdf]

size	Init. assign. no.	DFAsolution					
K=6		$\alpha = 0$	$\alpha = 0.1$	$\alpha = 0.3$	$\alpha = 0.5$	$\alpha = 0.7$	$\alpha = 1$
	1	50.971%	52.527%	53.048%	55.618%	55.289%	57.867%
	2	50.939%	50.895%	52.507%	54.336%	55.936%	57.923%
	3	49.451%	50.331%	52.352%	55.624%	56.744%	57.788%
	4	49.828%	52.194%	52.804%	55.419%	56.680%	58.604%
	5	50.987%	51.459%	53.029%	54.610%	55.638%	58.441%
Mean		50.435%	51.481%	52.748%	55.121%	56.057%	58.125%
SE Mean		0.003	0.004	0.001	0.002	0.002	0.001
S.D.		0.007	0.009	0.003	0.006	0.006	0.003
		$\alpha = 0$	$\alpha = 0.1$	$\alpha = 0.3$	$\alpha = 0.5$	$\alpha = 0.7$	$\alpha = 1$
K=7	1	53.469%	54.087%	54.431%	56.192%	58.116%	57.534%
	2	52.830%	54.220%	55.039%	57.179%	58.503%	59.046%
	3	53.923%	53.005%	54.424%	57.781%	56.723%	58.635%
	4	53.580%	53.969%	53.859%	56.617%	57.617%	59.180%
	5	53.282%	53.656%	55.539%	54.825%	57.236%	59.041%
Mean		53.417%	53.787%	54.658%	56.519%	57.639%	58.687%
SE Mean		0.001	0.002	0.002	0.005	0.003	0.003
S.D.		0.004	0.004	0.006	0.011	0.007	0.006
		$\alpha = 0$	$\alpha = 0.1$	$\alpha = 0.3$	$\alpha = 0.5$	$\alpha = 0.7$	$\alpha = 1$
K=8	1	53.160%	53.692%	54.438%	57.332%	59.896%	61.281%
	2	51.837%	56.064%	57.462%	57.531%	59.483%	62.910%
	3	52.511%	52.538%	55.797%	58.439%	59.061%	62.089%
	4	53.437%	53.928%	57.652%	58.694%	59.821%	60.880%
	5	53.823%	54.777%	54.765%	58.648%	58.930%	60.379%
Mean		52.954%	54.200%	56.023%	58.129%	59.438%	61.508%
SE Mean		0.003	0.005	0.006	0.002	0.001	0.004
S.D.		0.007	0.013	0.014	0.006	0.004	0.010
		$\alpha = 0$	$\alpha = 0.1$	$\alpha = 0.3$	$\alpha = 0.5$	$\alpha = 0.7$	$\alpha = 1$
K=9	1	53.268%	56.171%	56.016%	57.217%	60.927%	61.928%
	2	54.554%	56.412%	58.045%	59.243%	59.971%	61.676%
	3	52.942%	54.454%	58.006%	57.593%	58.478%	62.929%
	4	52.866%	55.073%	57.444%	58.143%	59.191%	62.821%
	5	52.616%	54.091%	56.257%	58.585%	60.941%	62.020%
Mean		53.249%	55.240%	57.154%	58.156%	59.902%	62.275%
SE Mean		0.003	0.004	0.004	0.003	0.004	0.002
S.D.		0.007	0.010	0.009	0.008	0.010	0.005
		$\alpha = 0$	$\alpha = 0.1$	$\alpha = 0.3$	$\alpha = 0.5$	$\alpha = 0.7$	$\alpha = 1$
K=10	1	54.988%	55.175%	56.752%	59.166%	60.544%	63.510%
	2	52.911%	52.341%	56.883%	58.985%	61.030%	62.007%
	3	53.477%	53.014%	57.139%	58.885%	59.531%	63.767%
	4	53.942%	54.696%	57.481%	59.369%	60.470%	63.151%
	5	51.117%	55.491%	55.189%	58.697%	61.679%	63.087%
Mean		53.287%	54.143%	56.689%	59.020%	60.651%	63.104%
SE Mean		0.006	0.006	0.003	0.001	0.003	0.003
S.D.		0.014	0.013	0.008	0.002	0.007	0.006
