# Supplementary material for: A portfolio selection model based on the knapsack problem under uncertainty
Source: PLoS One. 2019 May 1;14(5):e0213652. doi: 10.1371/journal.pone.0213652 (PMC6493714; doi:10.1371/journal.pone.0213652)
Supplement: S5 Table — (PDF) [file pone.0213652.s006.pdf]

		Mean solution of DFA	GAMS solution	S.D.	SE Mean	P-Value*
K=6	$\alpha = 0$	50.435%	49.234%	0.008	0.006	0.008
	$\alpha = 0.1$	51.481%	50.679%	0.005	0.004	0.005
	$\alpha = 0.3$	52.748%	53.378%	0.004	0.003	0.004
	$\alpha = 0.5$	55.121%	55.782%	0.004	0.003	0.004
	$\alpha = 0.7$	56.057%	57.744%	0.011	0.008	0.010
	$\alpha = 1$	58.125%	60.813%	0.019	0.013	0.015
K=7	$\alpha = 0$	53.417%	46.974%	0.045	0.032	0.042
	$\alpha = 0.1$	53.787%	49.254%	0.032	0.022	0.029
	$\alpha = 0.3$	54.658%	52.455%	0.015	0.011	0.013
	$\alpha = 0.5$	56.519%	54.811%	0.012	0.008	0.010
	$\alpha = 0.7$	57.639%	57.038%	0.004	0.003	0.003
	$\alpha = 1$	58.687%	59.925%	0.008	0.006	0.007
K=8	$\alpha = 0$	52.954%	45.652%	0.051	0.036	0.048
	$\alpha = 0.1$	54.200%	46.860%	0.051	0.036	0.047
	$\alpha = 0.3$	56.023%	49.489%	0.046	0.032	0.040
	$\alpha = 0.5$	58.129%	53.303%	0.034	0.024	0.028
	$\alpha = 0.7$	59.438%	55.749%	0.026	0.018	0.021
	$\alpha = 1$	61.508%	58.682%	0.020	0.014	0.015
K=9	$\alpha = 0$	53.249%	43.762%	0.067	0.047	0.063
	$\alpha = 0.1$	55.240%	45.431%	0.069	0.049	0.063
	$\alpha = 0.3$	57.154%	47.906%	0.065	0.046	0.057
	$\alpha = 0.5$	58.156%	50.061%	0.057	0.040	0.048
	$\alpha = 0.7$	59.902%	52.480%	0.052	0.037	0.043
	$\alpha = 1$	62.275%	57.077%	0.036	0.026	0.028
K=10	$\alpha = 0$	53.287%	36.058%	0.121	0.086	0.124
	$\alpha = 0.1$	54.143%	40.326%	0.097	0.069	0.094
	$\alpha = 0.3$	56.689%	44.145%	0.088	0.062	0.080
	$\alpha = 0.5$	59.020%	47.032%	0.084	0.059	0.073
	$\alpha = 0.7$	60.651%	49.702%	0.077	0.054	0.064
	$\alpha = 1$	63.104%	52.426%	0.075	0.053	0.060
