# Supplementary material for: A portfolio selection model based on the knapsack problem under uncertainty
Source: PLoS One. 2019 May 1;14(5):e0213652. doi: 10.1371/journal.pone.0213652 (PMC6493714; doi:10.1371/journal.pone.0213652)
Supplement: S6 Table — (PDF) [file pone.0213652.s007.pdf]

	D	DFA Solution						GAMS Solution					
		$\alpha = 0$	$\alpha = 0.1$	$\alpha = 0.3$	$\alpha = 0.5$	$\alpha = 0.7$	$\alpha = 1$	$\alpha = 0$	$\alpha = 0.1$	$\alpha = 0.3$	$\alpha = 0.5$	$\alpha = 0.7$	$\alpha = 1$
Low -D	K=6	50.435%	51.481%	52.748%	55.121%	56.057%	58.125%	49.234%	50.679%	53.378%	55.782%	57.744%	60.813%
	K=7	53.417%	53.787%	54.658%	56.519%	57.639%	58.687%	46.974%	49.254%	52.455%	54.811%	57.038%	59.925%
	K=8	52.954%	54.200%	56.023%	58.129%	59.438%	61.508%	45.652%	46.860%	49.489%	53.303%	55.749%	58.682%
	K=9	53.249%	55.240%	57.154%	58.156%	59.902%	62.275%	36.058%	40.326%	44.145%	47.032%	49.702%	52.426%
	K=10	53.287%	54.143%	56.689%	59.020%	60.651%	63.104%	36.058%	40.326%	44.145%	47.032%	49.702%	52.426%
High- D	K=11	52.664%	55.066%	56.087%	59.616%	62.251%	63.694%	-	-	-	-	-	-
	K=12	50.432%	52.244%	55.373%	58.155%	60.899%	63.898%	-	-	-	-	-	-
	K=13	48.351%	49.467%	52.614%	55.060%	57.667%	61.688%	-	-	-	-	-	-
	K=14	47.935%	48.951%	49.584%	52.767%	55.157%	57.829%	-	-	-	-	-	-
	K=15	44.075%	45.247%	47.652%	49.948%	52.576%	55.531%	-	-	-	-	-	-
