# Supplementary material for: A portfolio selection model based on the knapsack problem under uncertainty
Source: PLoS One. 2019 May 1;14(5):e0213652. doi: 10.1371/journal.pone.0213652 (PMC6493714; doi:10.1371/journal.pone.0213652)
Supplement: S1 Algorithm — (PDF) [file pone.0213652.s008.pdf]

Algorithm 1. Pseudocode of DFA

Suppose that $f(X)$ is the objective function of $X = \left(x_1, x_2, ..., x_d\right)^T$.
Assign value for β_0, φ, γ and $MaxGeneration.$
Generate initial population of fireflies x_i for $i = 1, 2, ..., n.$
Determine the light intensify I_i at x_i using $f(x_i)$.
while $(t < MaxGeneration)$ **do**
for $i = 1:n$ all n fireflies **do**
for $i = 1:n$ all n fireflies **do**
if $(I_i < I_j)$ **then**
Move firefly i towards j
End if
Vary attractiveness with distance r using $\exp\left(-\gamma r^2\right)$
Discrete the position of $i-th$ firefly (31)
Evaluate new solution (position of $i-th$ firefly) and update light intensify I_i
End for
End for
Rank the fireflies and find the current global best
End while
Show result and visualization.
