# Supplementary material for: A portfolio selection model based on the knapsack problem under uncertainty
Source: PLoS One. 2019 May 1;14(5):e0213652. doi: 10.1371/journal.pone.0213652 (PMC6493714; doi:10.1371/journal.pone.0213652)
Supplement: S2 Appendix — Survey form. (PDF) [file pone.0213652.s010.pdf]

Survey questionnaire for portfolio selection model under uncertainty

Dear Madam/Sir

This is an academic research about “portfolio selection model under uncertainty”. The purpose is to select the best portfolio with maximum returns and minimum risks. As a person familiar with the Dow Jones Industrial Average system, you must specify your preferences in choosing each stock against other stocks.
We would like to cordially invite you to contribute to our academic research by filling out the enclosed questionnaire. Having the honor of obtaining your precious opinions, the result and credibility of this research will be tremendously benefited. All the information provided will be used for academic purposes only, and will not be separately announced to the outside or transferred to other applications. Please feel at ease in filling out the answers.
Your support will be very crucial to the successful completion of this research. We sincerely hope that you would spend some time to express your opinions to be taken as reference for this research. Please accept our most sincere appreciation. Thank you and wish you all the best.

1. Instructions for filling out the questionnaire

The structure of this questionnaire is as follows. In section 2, information on the stocks of Dow Jones Industrial Average is presented. In Section 3, fundamental scale for pairwise comparisons is presented. Method for filling out is presented in Section 4. Finally, fill in the questionnaire by you in Section 5.

2. Descriptions of stocks of Dow Jones Industrial Average

Table 1 presents information on the symbols and the names of companies in the Dow Jones Industrial Index.

Table 1.Campany name.

	symbol	Company name
1	DWDP	DowDuPont Inc.
2	GE	General Electric Company
3	BA	The Boeing Company
4	AAPL	Apple Inc.
5	AXP	American Express Company
6	XOM	Exxon Mobil Corporation
7	MCD	McDonald's Corporation
8	DIS	The Walt Disney Company
9	TRV	The Travelers Companies, Inc.
10	WMT	Wal-Mart Stores, Inc.
11	CVX	Chevron Corporation
12	PG	The Procter & Gamble Company
13	UTX	United Technologies Corporation
14	JNJ	Johnson & Johnson
15	CSCO	Cisco Systems, Inc.
16	INTC	Intel Corporation
17	PFE	Pfizer Inc.
18	CAT	Caterpillar Inc.
19	GS	The Goldman Sachs Group, Inc.
20	HD	The Home Depot, Inc.
21	IBM	International Business Machines Corporation.
22	MMM	3M Company

23	KO	The Coca-Cola Company
24	V	Visa Inc.
25	NKE	NIKE, Inc.
26	JPM	JPMorgan Chase & Co.
27	VZ	Verizon Communications Inc.
28	MRK	Merck & Co., Inc.
29	UNH	UnitedHealth Group Incorporated
30	MSFT	Microsoft Corporation

3. Fundamental scale for pairwise comparisons

Table 2 shows the fundamental scale for pairwise comparisons.

Table 2. Fundamental scale for pairwise comparison.

Intensity of Importance	Definition	Explanation
1	Equal importance	Two elements contribute equally to the objective
3	Moderate importance	Experience and judgment slightly favor one element over another
5	Strong importance	Experience and judgment strongly favor one element over another
7	Very strong importance	One element is favored very strongly over another; its dominance is demonstrated in practice
9	Extreme importance	The evidence favoring one element over another is of the highest possible order of affirmation
Intensities of 2, 4, 6, and 8 can be used to express intermediate values. Intensities 1.1, 1.2, 1.3, etc can be used for elements that are very close in importance.		

4. Method for filling out level of importance and preference

Our model is an interval model. The importance of each stock to the other stock is determined by intervals numbers. Method for filling out the survey is described below with illustration.

Example 1:

If the criterion on the left is more important than the one on the right, the numbers on the left are selected (in the form of interval numbers).

**DD**	9	8	7	6	5	4	3	2	1	2	3	4	5	6	7	8	9	**BA**

These indicate that the importance of **DD** stock is higher than **BA** stock and its importance is in **[3,5]**.

OR:

If the criterion on the right is more important than the one on the left, the numbers on the right are selected (in the form of interval numbers).

**DD**	9	8	7	6	5	4	3	2	1	2	3	4	5	6	7	8	9	**BA**

These indicate that the importance of **BA** stock is higher than **DD** stock and its importance is in **[3,5]**.

Example 2:

**XOM**	9	8	7	6	5	4	3	2	1	2	3	4	5	6	7	8	9	**AAPL**

These indicate that the importance of **XOM** stock is higher than **AAPL** stock and its importance is in **[1, 2]**.

**XOM**	9	8	7	6	5	4	3	2	1	2	3	4	5	6	7	8	9	**AAPL**

These indicate that the importance of **AAPL** stock is higher than **XOM** stock and its importance is in **[1, 2]**.

5. The level of importance

Please fill out the level of importance.

**DD**	9	8	7	6	5	4	3	2	1	2	3	4	5	6	7	8	9	**GE**
**DD**	9	8	7	6	5	4	3	2	1	2	3	4	5	6	7	8	9	**BA**
**DD**	9	8	7	6	5	4	3	2	1	2	3	4	5	6	7	8	9	**AAPL**
**DD**	9	8	7	6	5	4	3	2	1	2	3	4	5	6	7	8	9	**AXP**
**DD**	9	8	7	6	5	4	3	2	1	2	3	4	5	6	7	8	9	**XOM**
**DD**	9	8	7	6	5	4	3	2	1	2	3	4	5	6	7	8	9	**MCD**
**DD**	9	8	7	6	5	4	3	2	1	2	3	4	5	6	7	8	9	**DIS**
**DD**	9	8	7	6	5	4	3	2	1	2	3	4	5	6	7	8	9	**TRV**
**DD**	9	8	7	6	5	4	3	2	1	2	3	4	5	6	7	8	9	**WMT**
**DD**	9	8	7	6	5	4	3	2	1	2	3	4	5	6	7	8	9	**CVX**
**DD**	9	8	7	6	5	4	3	2	1	2	3	4	5	6	7	8	9	**PG**
**DD**	9	8	7	6	5	4	3	2	1	2	3	4	5	6	7	8	9	**UTX**
**DD**	9	8	7	6	5	4	3	2	1	2	3	4	5	6	7	8	9	**JNJ**
**DD**	9	8	7	6	5	4	3	2	1	2	3	4	5	6	7	8	9	**CSCO**
**DD**	9	8	7	6	5	4	3	2	1	2	3	4	5	6	7	8	9	**INTC**

**DD**	9	8	7	6	5	4	3	2	1	2	3	4	5	6	7	8	9	**PFE**
**DD**	9	8	7	6	5	4	3	2	1	2	3	4	5	6	7	8	9	**CAT**
**DD**	9	8	7	6	5	4	3	2	1	2	3	4	5	6	7	8	9	**GS**
**DD**	9	8	7	6	5	4	3	2	1	2	3	4	5	6	7	8	9	**HD**
**DD**	9	8	7	6	5	4	3	2	1	2	3	4	5	6	7	8	9	**IBM**
**DD**	9	8	7	6	5	4	3	2	1	2	3	4	5	6	7	8	9	**MMM**
**DD**	9	8	7	6	5	4	3	2	1	2	3	4	5	6	7	8	9	**KO**
**DD**	9	8	7	6	5	4	3	2	1	2	3	4	5	6	7	8	9	**V**
**DD**	9	8	7	6	5	4	3	2	1	2	3	4	5	6	7	8	9	**NKE**
**DD**	9	8	7	6	5	4	3	2	1	2	3	4	5	6	7	8	9	**JPM**
**DD**	9	8	7	6	5	4	3	2	1	2	3	4	5	6	7	8	9	**VZ**
**DD**	9	8	7	6	5	4	3	2	1	2	3	4	5	6	7	8	9	**MRK**
**DD**	9	8	7	6	5	4	3	2	1	2	3	4	5	6	7	8	9	**UNH**
**DD**	9	8	7	6	5	4	3	2	1	2	3	4	5	6	7	8	9	**MSFT**

**GE**	9	8	7	6	5	4	3	2	1	2	3	4	5	6	7	8	9	BA
**GE**	9	8	7	6	5	4	3	2	1	2	3	4	5	6	7	8	9	AAPL
**GE**	9	8	7	6	5	4	3	2	1	2	3	4	5	6	7	8	9	AXP
**GE**	9	8	7	6	5	4	3	2	1	2	3	4	5	6	7	8	9	XOM
**GE**	9	8	7	6	5	4	3	2	1	2	3	4	5	6	7	8	9	MCD
**GE**	9	8	7	6	5	4	3	2	1	2	3	4	5	6	7	8	9	DIS
**GE**	9	8	7	6	5	4	3	2	1	2	3	4	5	6	7	8	9	TRV
**GE**	9	8	7	6	5	4	3	2	1	2	3	4	5	6	7	8	9	WMT
**GE**	9	8	7	6	5	4	3	2	1	2	3	4	5	6	7	8	9	**CVX**

**GE**	9	8	7	6	5	4	3	2	1	2	3	4	5	6	7	8	9	**PG**
**GE**	9	8	7	6	5	4	3	2	1	2	3	4	5	6	7	8	9	**UTX**
**GE**	9	8	7	6	5	4	3	2	1	2	3	4	5	6	7	8	9	**JNJ**
**GE**	9	8	7	6	5	4	3	2	1	2	3	4	5	6	7	8	9	**CSCO**
**GE**	9	8	7	6	5	4	3	2	1	2	3	4	5	6	7	8	9	**INTC**
**GE**	9	8	7	6	5	4	3	2	1	2	3	4	5	6	7	8	9	**PFE**
**GE**	9	8	7	6	5	4	3	2	1	2	3	4	5	6	7	8	9	**CAT**
**GE**	9	8	7	6	5	4	3	2	1	2	3	4	5	6	7	8	9	**GS**
**GE**	9	8	7	6	5	4	3	2	1	2	3	4	5	6	7	8	9	**HD**
**GE**	9	8	7	6	5	4	3	2	1	2	3	4	5	6	7	8	9	**IBM**
**GE**	9	8	7	6	5	4	3	2	1	2	3	4	5	6	7	8	9	**MMM**
**GE**	9	8	7	6	5	4	3	2	1	2	3	4	5	6	7	8	9	**KO**
**GE**	9	8	7	6	5	4	3	2	1	2	3	4	5	6	7	8	9	**V**
**GE**	9	8	7	6	5	4	3	2	1	2	3	4	5	6	7	8	9	**NKE**
**GE**	9	8	7	6	5	4	3	2	1	2	3	4	5	6	7	8	9	**JPM**
**GE**	9	8	7	6	5	4	3	2	1	2	3	4	5	6	7	8	9	**VZ**
**GE**	9	8	7	6	5	4	3	2	1	2	3	4	5	6	7	8	9	**MRK**
**GE**	9	8	7	6	5	4	3	2	1	2	3	4	5	6	7	8	9	**UNH**
**GE**	9	8	7	6	5	4	3	2	1	2	3	4	5	6	7	8	9	**MSFT**

**BA**	9	8	7	6	5	4	3	2	1	2	3	4	5	6	7	8	9	**AAPL**
**BA**	9	8	7	6	5	4	3	2	1	2	3	4	5	6	7	8	9	**AXP**
**BA**	9	8	7	6	5	4	3	2	1	2	3	4	5	6	7	8	9	**XOM**
**BA**	9	8	7	6	5	4	3	2	1	2	3	4	5	6	7	8	9	**MCD**

**BA**	9	8	7	6	5	4	3	2	1	2	3	4	5	6	7	8	9	**DIS**
**BA**	9	8	7	6	5	4	3	2	1	2	3	4	5	6	7	8	9	**TRV**
**BA**	9	8	7	6	5	4	3	2	1	2	3	4	5	6	7	8	9	**WMT**
**BA**	9	8	7	6	5	4	3	2	1	2	3	4	5	6	7	8	9	**CVX**
**BA**	9	8	7	6	5	4	3	2	1	2	3	4	5	6	7	8	9	**PG**
**BA**	9	8	7	6	5	4	3	2	1	2	3	4	5	6	7	8	9	**UTX**
**BA**	9	8	7	6	5	4	3	2	1	2	3	4	5	6	7	8	9	**JNJ**
**BA**	9	8	7	6	5	4	3	2	1	2	3	4	5	6	7	8	9	**CSCO**
**BA**	9	8	7	6	5	4	3	2	1	2	3	4	5	6	7	8	9	**INTC**
**BA**	9	8	7	6	5	4	3	2	1	2	3	4	5	6	7	8	9	**PFE**
**BA**	9	8	7	6	5	4	3	2	1	2	3	4	5	6	7	8	9	**CAT**
**BA**	9	8	7	6	5	4	3	2	1	2	3	4	5	6	7	8	9	**GS**
**BA**	9	8	7	6	5	4	3	2	1	2	3	4	5	6	7	8	9	**HD**
**BA**	9	8	7	6	5	4	3	2	1	2	3	4	5	6	7	8	9	**IBM**
**BA**	9	8	7	6	5	4	3	2	1	2	3	4	5	6	7	8	9	**MMM**
**BA**	9	8	7	6	5	4	3	2	1	2	3	4	5	6	7	8	9	**KO**
**BA**	9	8	7	6	5	4	3	2	1	2	3	4	5	6	7	8	9	**V**
**BA**	9	8	7	6	5	4	3	2	1	2	3	4	5	6	7	8	9	**NKE**
**BA**	9	8	7	6	5	4	3	2	1	2	3	4	5	6	7	8	9	**JPM**
**BA**	9	8	7	6	5	4	3	2	1	2	3	4	5	6	7	8	9	**VZ**
**BA**	9	8	7	6	5	4	3	2	1	2	3	4	5	6	7	8	9	**MRK**
**BA**	9	8	7	6	5	4	3	2	1	2	3	4	5	6	7	8	9	**UNH**
**BA**	9	8	7	6	5	4	3	2	1	2	3	4	5	6	7	8	9	**MSFT**

**AAPL**	9	8	7	6	5	4	3	2	1	2	3	4	5	6	7	8	9	AXP
**AAPL**	9	8	7	6	5	4	3	2	1	2	3	4	5	6	7	8	9	XOM
**AAPL**	9	8	7	6	5	4	3	2	1	2	3	4	5	6	7	8	9	MCD
**AAPL**	9	8	7	6	5	4	3	2	1	2	3	4	5	6	7	8	9	DIS
**AAPL**	9	8	7	6	5	4	3	2	1	2	3	4	5	6	7	8	9	TRV
**AAPL**	9	8	7	6	5	4	3	2	1	2	3	4	5	6	7	8	9	WMT
**AAPL**	9	8	7	6	5	4	3	2	1	2	3	4	5	6	7	8	9	**CVX**
**AAPL**	9	8	7	6	5	4	3	2	1	2	3	4	5	6	7	8	9	**PG**
**AAPL**	9	8	7	6	5	4	3	2	1	2	3	4	5	6	7	8	9	**UTX**
**AAPL**	9	8	7	6	5	4	3	2	1	2	3	4	5	6	7	8	9	**JNJ**
**AAPL**	9	8	7	6	5	4	3	2	1	2	3	4	5	6	7	8	9	**CSCO**
**AAPL**	9	8	7	6	5	4	3	2	1	2	3	4	5	6	7	8	9	**INTC**
**AAPL**	9	8	7	6	5	4	3	2	1	2	3	4	5	6	7	8	9	**PFE**
**AAPL**	9	8	7	6	5	4	3	2	1	2	3	4	5	6	7	8	9	**CAT**
**AAPL**	9	8	7	6	5	4	3	2	1	2	3	4	5	6	7	8	9	**GS**
**AAPL**	9	8	7	6	5	4	3	2	1	2	3	4	5	6	7	8	9	**HD**
**AAPL**	9	8	7	6	5	4	3	2	1	2	3	4	5	6	7	8	9	**IBM**
**AAPL**	9	8	7	6	5	4	3	2	1	2	3	4	5	6	7	8	9	**MMM**
**AAPL**	9	8	7	6	5	4	3	2	1	2	3	4	5	6	7	8	9	**KO**
**AAPL**	9	8	7	6	5	4	3	2	1	2	3	4	5	6	7	8	9	**V**
**AAPL**	9	8	7	6	5	4	3	2	1	2	3	4	5	6	7	8	9	**NKE**
**AAPL**	9	8	7	6	5	4	3	2	1	2	3	4	5	6	7	8	9	**JPM**
**AAPL**	9	8	7	6	5	4	3	2	1	2	3	4	5	6	7	8	9	**VZ**
**AAPL**	9	8	7	6	5	4	3	2	1	2	3	4	5	6	7	8	9	**MRK**

**AAPL**	9	8	7	6	5	4	3	2	1	2	3	4	5	6	7	8	9	**UNH**
**AAPL**	9	8	7	6	5	4	3	2	1	2	3	4	5	6	7	8	9	**MSFT**

**AXP**	9	8	7	6	5	4	3	2	1	2	3	4	5	6	7	8	9	XOM
**AXP**	9	8	7	6	5	4	3	2	1	2	3	4	5	6	7	8	9	MCD
**AXP**	9	8	7	6	5	4	3	2	1	2	3	4	5	6	7	8	9	DIS
**AXP**	9	8	7	6	5	4	3	2	1	2	3	4	5	6	7	8	9	TRV
**AXP**	9	8	7	6	5	4	3	2	1	2	3	4	5	6	7	8	9	WMT
**AXP**	9	8	7	6	5	4	3	2	1	2	3	4	5	6	7	8	9	**CVX**
**AXP**	9	8	7	6	5	4	3	2	1	2	3	4	5	6	7	8	9	**PG**
**AXP**	9	8	7	6	5	4	3	2	1	2	3	4	5	6	7	8	9	**UTX**
**AXP**	9	8	7	6	5	4	3	2	1	2	3	4	5	6	7	8	9	**JNJ**
**AXP**	9	8	7	6	5	4	3	2	1	2	3	4	5	6	7	8	9	**CSCO**
**AXP**	9	8	7	6	5	4	3	2	1	2	3	4	5	6	7	8	9	**INTC**
**AXP**	9	8	7	6	5	4	3	2	1	2	3	4	5	6	7	8	9	**PFE**
**AXP**	9	8	7	6	5	4	3	2	1	2	3	4	5	6	7	8	9	**CAT**
**AXP**	9	8	7	6	5	4	3	2	1	2	3	4	5	6	7	8	9	**GS**
**AXP**	9	8	7	6	5	4	3	2	1	2	3	4	5	6	7	8	9	**HD**
**AXP**	9	8	7	6	5	4	3	2	1	2	3	4	5	6	7	8	9	**IBM**
**AXP**	9	8	7	6	5	4	3	2	1	2	3	4	5	6	7	8	9	**MMM**
**AXP**	9	8	7	6	5	4	3	2	1	2	3	4	5	6	7	8	9	**KO**
**AXP**	9	8	7	6	5	4	3	2	1	2	3	4	5	6	7	8	9	**V**
**AXP**	9	8	7	6	5	4	3	2	1	2	3	4	5	6	7	8	9	**NKE**
**AXP**	9	8	7	6	5	4	3	2	1	2	3	4	5	6	7	8	9	**JPM**

**AXP**	9	8	7	6	5	4	3	2	1	2	3	4	5	6	7	8	9	**VZ**
**AXP**	9	8	7	6	5	4	3	2	1	2	3	4	5	6	7	8	9	**MRK**
**AXP**	9	8	7	6	5	4	3	2	1	2	3	4	5	6	7	8	9	**UNH**
**AXP**	9	8	7	6	5	4	3	2	1	2	3	4	5	6	7	8	9	**MSFT**

**XOM**	9	8	7	6	5	4	3	2	1	2	3	4	5	6	7	8	9	**MCD**
**XOM**	9	8	7	6	5	4	3	2	1	2	3	4	5	6	7	8	9	**DIS**
**XOM**	9	8	7	6	5	4	3	2	1	2	3	4	5	6	7	8	9	**TRV**
**XOM**	9	8	7	6	5	4	3	2	1	2	3	4	5	6	7	8	9	**WMT**
**XOM**	9	8	7	6	5	4	3	2	1	2	3	4	5	6	7	8	9	**CVX**
**XOM**	9	8	7	6	5	4	3	2	1	2	3	4	5	6	7	8	9	**PG**
**XOM**	9	8	7	6	5	4	3	2	1	2	3	4	5	6	7	8	9	**UTX**
**XOM**	9	8	7	6	5	4	3	2	1	2	3	4	5	6	7	8	9	**JNJ**
**XOM**	9	8	7	6	5	4	3	2	1	2	3	4	5	6	7	8	9	**CSCO**
**XOM**	9	8	7	6	5	4	3	2	1	2	3	4	5	6	7	8	9	**INTC**
**XOM**	9	8	7	6	5	4	3	2	1	2	3	4	5	6	7	8	9	**PFE**
**XOM**	9	8	7	6	5	4	3	2	1	2	3	4	5	6	7	8	9	**CAT**
**XOM**	9	8	7	6	5	4	3	2	1	2	3	4	5	6	7	8	9	**GS**
**XOM**	9	8	7	6	5	4	3	2	1	2	3	4	5	6	7	8	9	**HD**
**XOM**	9	8	7	6	5	4	3	2	1	2	3	4	5	6	7	8	9	**IBM**
**XOM**	9	8	7	6	5	4	3	2	1	2	3	4	5	6	7	8	9	**MMM**
**XOM**	9	8	7	6	5	4	3	2	1	2	3	4	5	6	7	8	9	**KO**
**XOM**	9	8	7	6	5	4	3	2	1	2	3	4	5	6	7	8	9	**V**
**XOM**	9	8	7	6	5	4	3	2	1	2	3	4	5	6	7	8	9	**NKE**

**XOM**	9	8	7	6	5	4	3	2	1	2	3	4	5	6	7	8	9	**JPM**
**XOM**	9	8	7	6	5	4	3	2	1	2	3	4	5	6	7	8	9	**VZ**
**XOM**	9	8	7	6	5	4	3	2	1	2	3	4	5	6	7	8	9	**MRK**
**XOM**	9	8	7	6	5	4	3	2	1	2	3	4	5	6	7	8	9	**UNH**
**XOM**	9	8	7	6	5	4	3	2	1	2	3	4	5	6	7	8	9	**MSFT**

**MCD**	9	8	7	6	5	4	3	2	1	2	3	4	5	6	7	8	9	DIS
**MCD**	9	8	7	6	5	4	3	2	1	2	3	4	5	6	7	8	9	TRV
**MCD**	9	8	7	6	5	4	3	2	1	2	3	4	5	6	7	8	9	WMT
**MCD**	9	8	7	6	5	4	3	2	1	2	3	4	5	6	7	8	9	**CVX**
**MCD**	9	8	7	6	5	4	3	2	1	2	3	4	5	6	7	8	9	**PG**
**MCD**	9	8	7	6	5	4	3	2	1	2	3	4	5	6	7	8	9	**UTX**
**MCD**	9	8	7	6	5	4	3	2	1	2	3	4	5	6	7	8	9	**JNJ**
**MCD**	9	8	7	6	5	4	3	2	1	2	3	4	5	6	7	8	9	**CSCO**
**MCD**	9	8	7	6	5	4	3	2	1	2	3	4	5	6	7	8	9	**INTC**
**MCD**	9	8	7	6	5	4	3	2	1	2	3	4	5	6	7	8	9	**PFE**
**MCD**	9	8	7	6	5	4	3	2	1	2	3	4	5	6	7	8	9	**CAT**
**MCD**	9	8	7	6	5	4	3	2	1	2	3	4	5	6	7	8	9	**GS**
**MCD**	9	8	7	6	5	4	3	2	1	2	3	4	5	6	7	8	9	**HD**
**MCD**	9	8	7	6	5	4	3	2	1	2	3	4	5	6	7	8	9	**IBM**
**MCD**	9	8	7	6	5	4	3	2	1	2	3	4	5	6	7	8	9	**MMM**
**MCD**	9	8	7	6	5	4	3	2	1	2	3	4	5	6	7	8	9	**KO**
**MCD**	9	8	7	6	5	4	3	2	1	2	3	4	5	6	7	8	9	**V**
**MCD**	9	8	7	6	5	4	3	2	1	2	3	4	5	6	7	8	9	**NKE**

**MCD**	9	8	7	6	5	4	3	2	1	2	3	4	5	6	7	8	9	**JPM**
**MCD**	9	8	7	6	5	4	3	2	1	2	3	4	5	6	7	8	9	**VZ**
**MCD**	9	8	7	6	5	4	3	2	1	2	3	4	5	6	7	8	9	**MRK**
**MCD**	9	8	7	6	5	4	3	2	1	2	3	4	5	6	7	8	9	**UNH**
**MCD**	9	8	7	6	5	4	3	2	1	2	3	4	5	6	7	8	9	**MSFT**

**DIS**	9	8	7	6	5	4	3	2	1	2	3	4	5	6	7	8	9	**TRV**
**DIS**	9	8	7	6	5	4	3	2	1	2	3	4	5	6	7	8	9	**WMT**
**DIS**	9	8	7	6	5	4	3	2	1	2	3	4	5	6	7	8	9	**CVX**
**DIS**	9	8	7	6	5	4	3	2	1	2	3	4	5	6	7	8	9	**PG**
**DIS**	9	8	7	6	5	4	3	2	1	2	3	4	5	6	7	8	9	**UTX**
**DIS**	9	8	7	6	5	4	3	2	1	2	3	4	5	6	7	8	9	**JNJ**
**DIS**	9	8	7	6	5	4	3	2	1	2	3	4	5	6	7	8	9	**CSCO**
**DIS**	9	8	7	6	5	4	3	2	1	2	3	4	5	6	7	8	9	**INTC**
**DIS**	9	8	7	6	5	4	3	2	1	2	3	4	5	6	7	8	9	**PFE**
**DIS**	9	8	7	6	5	4	3	2	1	2	3	4	5	6	7	8	9	**CAT**
**DIS**	9	8	7	6	5	4	3	2	1	2	3	4	5	6	7	8	9	**GS**
**DIS**	9	8	7	6	5	4	3	2	1	2	3	4	5	6	7	8	9	**HD**
**DIS**	9	8	7	6	5	4	3	2	1	2	3	4	5	6	7	8	9	**IBM**
**DIS**	9	8	7	6	5	4	3	2	1	2	3	4	5	6	7	8	9	**MMM**
**DIS**	9	8	7	6	5	4	3	2	1	2	3	4	5	6	7	8	9	**KO**
**DIS**	9	8	7	6	5	4	3	2	1	2	3	4	5	6	7	8	9	**V**
**DIS**	9	8	7	6	5	4	3	2	1	2	3	4	5	6	7	8	9	**NKE**
**DIS**	9	8	7	6	5	4	3	2	1	2	3	4	5	6	7	8	9	**JPM**

**DIS**	9	8	7	6	5	4	3	2	1	2	3	4	5	6	7	8	9	**VZ**
**DIS**	9	8	7	6	5	4	3	2	1	2	3	4	5	6	7	8	9	**MRK**
**DIS**	9	8	7	6	5	4	3	2	1	2	3	4	5	6	7	8	9	**UNH**
**DIS**	9	8	7	6	5	4	3	2	1	2	3	4	5	6	7	8	9	**MSFT**

**TRV**	9	8	7	6	5	4	3	2	1	2	3	4	5	6	7	8	9	**WMT**
**TRV**	9	8	7	6	5	4	3	2	1	2	3	4	5	6	7	8	9	**CVX**
**TRV**	9	8	7	6	5	4	3	2	1	2	3	4	5	6	7	8	9	**PG**
**TRV**	9	8	7	6	5	4	3	2	1	2	3	4	5	6	7	8	9	**UTX**
**TRV**	9	8	7	6	5	4	3	2	1	2	3	4	5	6	7	8	9	**JNJ**
**TRV**	9	8	7	6	5	4	3	2	1	2	3	4	5	6	7	8	9	**CSCO**
**TRV**	9	8	7	6	5	4	3	2	1	2	3	4	5	6	7	8	9	**INTC**
**TRV**	9	8	7	6	5	4	3	2	1	2	3	4	5	6	7	8	9	**PFE**
**TRV**	9	8	7	6	5	4	3	2	1	2	3	4	5	6	7	8	9	**CAT**
**TRV**	9	8	7	6	5	4	3	2	1	2	3	4	5	6	7	8	9	**GS**
**TRV**	9	8	7	6	5	4	3	2	1	2	3	4	5	6	7	8	9	**HD**
**TRV**	9	8	7	6	5	4	3	2	1	2	3	4	5	6	7	8	9	**IBM**
**TRV**	9	8	7	6	5	4	3	2	1	2	3	4	5	6	7	8	9	**MMM**
**TRV**	9	8	7	6	5	4	3	2	1	2	3	4	5	6	7	8	9	**KO**
**TRV**	9	8	7	6	5	4	3	2	1	2	3	4	5	6	7	8	9	**V**
**TRV**	9	8	7	6	5	4	3	2	1	2	3	4	5	6	7	8	9	**NKE**
**TRV**	9	8	7	6	5	4	3	2	1	2	3	4	5	6	7	8	9	**JPM**
**TRV**	9	8	7	6	5	4	3	2	1	2	3	4	5	6	7	8	9	**VZ**
**TRV**	9	8	7	6	5	4	3	2	1	2	3	4	5	6	7	8	9	**MRK**

**TRV**	9	8	7	6	5	4	3	2	1	2	3	4	5	6	7	8	9	**UNH**
**TRV**	9	8	7	6	5	4	3	2	1	2	3	4	5	6	7	8	9	**MSFT**

**WMT**	9	8	7	6	5	4	3	2	1	2	3	4	5	6	7	8	9	**CVX**
**WMT**	9	8	7	6	5	4	3	2	1	2	3	4	5	6	7	8	9	**PG**
**WMT**	9	8	7	6	5	4	3	2	1	2	3	4	5	6	7	8	9	**UTX**
**WMT**	9	8	7	6	5	4	3	2	1	2	3	4	5	6	7	8	9	**JNJ**
**WMT**	9	8	7	6	5	4	3	2	1	2	3	4	5	6	7	8	9	**CSCO**
**WMT**	9	8	7	6	5	4	3	2	1	2	3	4	5	6	7	8	9	**INTC**
**WMT**	9	8	7	6	5	4	3	2	1	2	3	4	5	6	7	8	9	**PFE**
**WMT**	9	8	7	6	5	4	3	2	1	2	3	4	5	6	7	8	9	**CAT**
**WMT**	9	8	7	6	5	4	3	2	1	2	3	4	5	6	7	8	9	**GS**
**WMT**	9	8	7	6	5	4	3	2	1	2	3	4	5	6	7	8	9	**HD**
**WMT**	9	8	7	6	5	4	3	2	1	2	3	4	5	6	7	8	9	**IBM**
**WMT**	9	8	7	6	5	4	3	2	1	2	3	4	5	6	7	8	9	**MMM**
**WMT**	9	8	7	6	5	4	3	2	1	2	3	4	5	6	7	8	9	**KO**
**WMT**	9	8	7	6	5	4	3	2	1	2	3	4	5	6	7	8	9	**V**
**WMT**	9	8	7	6	5	4	3	2	1	2	3	4	5	6	7	8	9	**NKE**
**WMT**	9	8	7	6	5	4	3	2	1	2	3	4	5	6	7	8	9	**JPM**
**WMT**	9	8	7	6	5	4	3	2	1	2	3	4	5	6	7	8	9	**VZ**
**WMT**	9	8	7	6	5	4	3	2	1	2	3	4	5	6	7	8	9	**MRK**
**WMT**	9	8	7	6	5	4	3	2	1	2	3	4	5	6	7	8	9	**UNH**
**WMT**	9	8	7	6	5	4	3	2	1	2	3	4	5	6	7	8	9	**MSFT**

**CVX**	9	8	7	6	5	4	3	2	1	2	3	4	5	6	7	8	9	**PG**
**CVX**	9	8	7	6	5	4	3	2	1	2	3	4	5	6	7	8	9	**UTX**
**CVX**	9	8	7	6	5	4	3	2	1	2	3	4	5	6	7	8	9	**JNJ**
**CVX**	9	8	7	6	5	4	3	2	1	2	3	4	5	6	7	8	9	**CSCO**
**CVX**	9	8	7	6	5	4	3	2	1	2	3	4	5	6	7	8	9	**INTC**
**CVX**	9	8	7	6	5	4	3	2	1	2	3	4	5	6	7	8	9	**PFE**
**CVX**	9	8	7	6	5	4	3	2	1	2	3	4	5	6	7	8	9	**CAT**
**CVX**	9	8	7	6	5	4	3	2	1	2	3	4	5	6	7	8	9	**GS**
**CVX**	9	8	7	6	5	4	3	2	1	2	3	4	5	6	7	8	9	**HD**
**CVX**	9	8	7	6	5	4	3	2	1	2	3	4	5	6	7	8	9	**IBM**
**CVX**	9	8	7	6	5	4	3	2	1	2	3	4	5	6	7	8	9	**MMM**
**CVX**	9	8	7	6	5	4	3	2	1	2	3	4	5	6	7	8	9	**KO**
**CVX**	9	8	7	6	5	4	3	2	1	2	3	4	5	6	7	8	9	**V**
**CVX**	9	8	7	6	5	4	3	2	1	2	3	4	5	6	7	8	9	**NKE**
**CVX**	9	8	7	6	5	4	3	2	1	2	3	4	5	6	7	8	9	**JPM**
**CVX**	9	8	7	6	5	4	3	2	1	2	3	4	5	6	7	8	9	**VZ**
**CVX**	9	8	7	6	5	4	3	2	1	2	3	4	5	6	7	8	9	**MRK**
**CVX**	9	8	7	6	5	4	3	2	1	2	3	4	5	6	7	8	9	**UNH**
**CVX**	9	8	7	6	5	4	3	2	1	2	3	4	5	6	7	8	9	**MSFT**

**PG**	9	8	7	6	5	4	3	2	1	2	3	4	5	6	7	8	9	**UTX**
**PG**	9	8	7	6	5	4	3	2	1	2	3	4	5	6	7	8	9	**JNJ**

**PG**	9	8	7	6	5	4	3	2	1	2	3	4	5	6	7	8	9	**CSCO**
**PG**	9	8	7	6	5	4	3	2	1	2	3	4	5	6	7	8	9	**INTC**
**PG**	9	8	7	6	5	4	3	2	1	2	3	4	5	6	7	8	9	**PFE**
**PG**	9	8	7	6	5	4	3	2	1	2	3	4	5	6	7	8	9	**CAT**
**PG**	9	8	7	6	5	4	3	2	1	2	3	4	5	6	7	8	9	**GS**
**PG**	9	8	7	6	5	4	3	2	1	2	3	4	5	6	7	8	9	**HD**
**PG**	9	8	7	6	5	4	3	2	1	2	3	4	5	6	7	8	9	**IBM**
**PG**	9	8	7	6	5	4	3	2	1	2	3	4	5	6	7	8	9	**MMM**
**PG**	9	8	7	6	5	4	3	2	1	2	3	4	5	6	7	8	9	**KO**
**PG**	9	8	7	6	5	4	3	2	1	2	3	4	5	6	7	8	9	**V**
**PG**	9	8	7	6	5	4	3	2	1	2	3	4	5	6	7	8	9	**NKE**
**PG**	9	8	7	6	5	4	3	2	1	2	3	4	5	6	7	8	9	**JPM**
**PG**	9	8	7	6	5	4	3	2	1	2	3	4	5	6	7	8	9	**VZ**
**PG**	9	8	7	6	5	4	3	2	1	2	3	4	5	6	7	8	9	**MRK**
**PG**	9	8	7	6	5	4	3	2	1	2	3	4	5	6	7	8	9	**UNH**
**PG**	9	8	7	6	5	4	3	2	1	2	3	4	5	6	7	8	9	**MSFT**

**UTX**	9	8	7	6	5	4	3	2	1	2	3	4	5	6	7	8	9	**JNJ**
**UTX**	9	8	7	6	5	4	3	2	1	2	3	4	5	6	7	8	9	**CSCO**
**UTX**	9	8	7	6	5	4	3	2	1	2	3	4	5	6	7	8	9	**INTC**
**UTX**	9	8	7	6	5	4	3	2	1	2	3	4	5	6	7	8	9	**PFE**
**UTX**	9	8	7	6	5	4	3	2	1	2	3	4	5	6	7	8	9	**CAT**
**UTX**	9	8	7	6	5	4	3	2	1	2	3	4	5	6	7	8	9	**GS**
**UTX**	9	8	7	6	5	4	3	2	1	2	3	4	5	6	7	8	9	**HD**
**UTX**	9	8	7	6	5	4	3	2	1	2	3	4	5	6	7	8	9	**IBM**

**UTX**	9	8	7	6	5	4	3	2	1	2	3	4	5	6	7	8	9	**MMM**
**UTX**	9	8	7	6	5	4	3	2	1	2	3	4	5	6	7	8	9	**KO**
**UTX**	9	8	7	6	5	4	3	2	1	2	3	4	5	6	7	8	9	**V**
**UTX**	9	8	7	6	5	4	3	2	1	2	3	4	5	6	7	8	9	**NKE**
**UTX**	9	8	7	6	5	4	3	2	1	2	3	4	5	6	7	8	9	**JPM**
**UTX**	9	8	7	6	5	4	3	2	1	2	3	4	5	6	7	8	9	**VZ**
**UTX**	9	8	7	6	5	4	3	2	1	2	3	4	5	6	7	8	9	**MRK**
**UTX**	9	8	7	6	5	4	3	2	1	2	3	4	5	6	7	8	9	**UNH**
**UTX**	9	8	7	6	5	4	3	2	1	2	3	4	5	6	7	8	9	**MSFT**

**JNJ**	9	8	7	6	5	4	3	2	1	2	3	4	5	6	7	8	9	**CSCO**
**JNJ**	9	8	7	6	5	4	3	2	1	2	3	4	5	6	7	8	9	**INTC**
**JNJ**	9	8	7	6	5	4	3	2	1	2	3	4	5	6	7	8	9	**PFE**
**JNJ**	9	8	7	6	5	4	3	2	1	2	3	4	5	6	7	8	9	**CAT**
**JNJ**	9	8	7	6	5	4	3	2	1	2	3	4	5	6	7	8	9	**GS**
**JNJ**	9	8	7	6	5	4	3	2	1	2	3	4	5	6	7	8	9	**HD**
**JNJ**	9	8	7	6	5	4	3	2	1	2	3	4	5	6	7	8	9	**IBM**
**JNJ**	9	8	7	6	5	4	3	2	1	2	3	4	5	6	7	8	9	**MMM**
**JNJ**	9	8	7	6	5	4	3	2	1	2	3	4	5	6	7	8	9	**KO**
**JNJ**	9	8	7	6	5	4	3	2	1	2	3	4	5	6	7	8	9	**V**
**JNJ**	9	8	7	6	5	4	3	2	1	2	3	4	5	6	7	8	9	**NKE**
**JNJ**	9	8	7	6	5	4	3	2	1	2	3	4	5	6	7	8	9	**JPM**
**JNJ**	9	8	7	6	5	4	3	2	1	2	3	4	5	6	7	8	9	**VZ**
**JNJ**	9	8	7	6	5	4	3	2	1	2	3	4	5	6	7	8	9	**MRK**
**JNJ**	9	8	7	6	5	4	3	2	1	2	3	4	5	6	7	8	9	**UNH**

**JNJ**	9	8	7	6	5	4	3	2	1	2	3	4	5	6	7	8	9	**MSFT**

**CSCO**	9	8	7	6	5	4	3	2	1	2	3	4	5	6	7	8	9	**INTC**
**CSCO**	9	8	7	6	5	4	3	2	1	2	3	4	5	6	7	8	9	**PFE**
**CSCO**	9	8	7	6	5	4	3	2	1	2	3	4	5	6	7	8	9	**CAT**
**CSCO**	9	8	7	6	5	4	3	2	1	2	3	4	5	6	7	8	9	**GS**
**CSCO**	9	8	7	6	5	4	3	2	1	2	3	4	5	6	7	8	9	**HD**
**CSCO**	9	8	7	6	5	4	3	2	1	2	3	4	5	6	7	8	9	**IBM**
**CSCO**	9	8	7	6	5	4	3	2	1	2	3	4	5	6	7	8	9	**MMM**
**CSCO**	9	8	7	6	5	4	3	2	1	2	3	4	5	6	7	8	9	**KO**
**CSCO**	9	8	7	6	5	4	3	2	1	2	3	4	5	6	7	8	9	**V**
**CSCO**	9	8	7	6	5	4	3	2	1	2	3	4	5	6	7	8	9	**NKE**
**CSCO**	9	8	7	6	5	4	3	2	1	2	3	4	5	6	7	8	9	**JPM**
**CSCO**	9	8	7	6	5	4	3	2	1	2	3	4	5	6	7	8	9	**VZ**
**CSCO**	9	8	7	6	5	4	3	2	1	2	3	4	5	6	7	8	9	**MRK**
**CSCO**	9	8	7	6	5	4	3	2	1	2	3	4	5	6	7	8	9	**UNH**
**CSCO**	9	8	7	6	5	4	3	2	1	2	3	4	5	6	7	8	9	**MSFT**

**INTC**	9	8	7	6	5	4	3	2	1	2	3	4	5	6	7	8	9	**PFE**
**INTC**	9	8	7	6	5	4	3	2	1	2	3	4	5	6	7	8	9	**CAT**
**INTC**	9	8	7	6	5	4	3	2	1	2	3	4	5	6	7	8	9	**GS**
**INTC**	9	8	7	6	5	4	3	2	1	2	3	4	5	6	7	8	9	**HD**
**INTC**	9	8	7	6	5	4	3	2	1	2	3	4	5	6	7	8	9	**IBM**
**INTC**	9	8	7	6	5	4	3	2	1	2	3	4	5	6	7	8	9	**MMM**

**INTC**	9	8	7	6	5	4	3	2	1	2	3	4	5	6	7	8	9	**KO**
**INTC**	9	8	7	6	5	4	3	2	1	2	3	4	5	6	7	8	9	**V**
**INTC**	9	8	7	6	5	4	3	2	1	2	3	4	5	6	7	8	9	**NKE**
**INTC**	9	8	7	6	5	4	3	2	1	2	3	4	5	6	7	8	9	**JPM**
**INTC**	9	8	7	6	5	4	3	2	1	2	3	4	5	6	7	8	9	**VZ**
**INTC**	9	8	7	6	5	4	3	2	1	2	3	4	5	6	7	8	9	**MRK**
**INTC**	9	8	7	6	5	4	3	2	1	2	3	4	5	6	7	8	9	**UNH**
**INTC**	9	8	7	6	5	4	3	2	1	2	3	4	5	6	7	8	9	**MSFT**

**PFE**	9	8	7	6	5	4	3	2	1	2	3	4	5	6	7	8	9	**CAT**
**PFE**	9	8	7	6	5	4	3	2	1	2	3	4	5	6	7	8	9	**GS**
**PFE**	9	8	7	6	5	4	3	2	1	2	3	4	5	6	7	8	9	**HD**
**PFE**	9	8	7	6	5	4	3	2	1	2	3	4	5	6	7	8	9	**IBM**
**PFE**	9	8	7	6	5	4	3	2	1	2	3	4	5	6	7	8	9	**MMM**
**PFE**	9	8	7	6	5	4	3	2	1	2	3	4	5	6	7	8	9	**KO**
**PFE**	9	8	7	6	5	4	3	2	1	2	3	4	5	6	7	8	9	**V**
**PFE**	9	8	7	6	5	4	3	2	1	2	3	4	5	6	7	8	9	**NKE**
**PFE**	9	8	7	6	5	4	3	2	1	2	3	4	5	6	7	8	9	**JPM**
**PFE**	9	8	7	6	5	4	3	2	1	2	3	4	5	6	7	8	9	**VZ**
**PFE**	9	8	7	6	5	4	3	2	1	2	3	4	5	6	7	8	9	**MRK**
**PFE**	9	8	7	6	5	4	3	2	1	2	3	4	5	6	7	8	9	**UNH**
**PFE**	9	8	7	6	5	4	3	2	1	2	3	4	5	6	7	8	9	**MSFT**

**CAT**	9	8	7	6	5	4	3	2	1	2	3	4	5	6	7	8	9	**GS**

**CAT**	9	8	7	6	5	4	3	2	1	2	3	4	5	6	7	8	9	**HD**
**CAT**	9	8	7	6	5	4	3	2	1	2	3	4	5	6	7	8	9	**IBM**
**CAT**	9	8	7	6	5	4	3	2	1	2	3	4	5	6	7	8	9	**MMM**
**CAT**	9	8	7	6	5	4	3	2	1	2	3	4	5	6	7	8	9	**KO**
**CAT**	9	8	7	6	5	4	3	2	1	2	3	4	5	6	7	8	9	**V**
**CAT**	9	8	7	6	5	4	3	2	1	2	3	4	5	6	7	8	9	**NKE**
**CAT**	9	8	7	6	5	4	3	2	1	2	3	4	5	6	7	8	9	**JPM**
**CAT**	9	8	7	6	5	4	3	2	1	2	3	4	5	6	7	8	9	**VZ**
**CAT**	9	8	7	6	5	4	3	2	1	2	3	4	5	6	7	8	9	**MRK**
**CAT**	9	8	7	6	5	4	3	2	1	2	3	4	5	6	7	8	9	**UNH**
**CAT**	9	8	7	6	5	4	3	2	1	2	3	4	5	6	7	8	9	**MSFT**

**GS**	9	8	7	6	5	4	3	2	1	2	3	4	5	6	7	8	9	**HD**
**GS**	9	8	7	6	5	4	3	2	1	2	3	4	5	6	7	8	9	**IBM**
**GS**	9	8	7	6	5	4	3	2	1	2	3	4	5	6	7	8	9	**MMM**
**GS**	9	8	7	6	5	4	3	2	1	2	3	4	5	6	7	8	9	**KO**
**GS**	9	8	7	6	5	4	3	2	1	2	3	4	5	6	7	8	9	**V**
**GS**	9	8	7	6	5	4	3	2	1	2	3	4	5	6	7	8	9	**NKE**
**GS**	9	8	7	6	5	4	3	2	1	2	3	4	5	6	7	8	9	**JPM**
**GS**	9	8	7	6	5	4	3	2	1	2	3	4	5	6	7	8	9	**VZ**
**GS**	9	8	7	6	5	4	3	2	1	2	3	4	5	6	7	8	9	**MRK**
**GS**	9	8	7	6	5	4	3	2	1	2	3	4	5	6	7	8	9	**UNH**
**GS**	9	8	7	6	5	4	3	2	1	2	3	4	5	6	7	8	9	**MSFT**

**HD**	9	8	7	6	5	4	3	2	1	2	3	4	5	6	7	8	9	**IBM**
**HD**	9	8	7	6	5	4	3	2	1	2	3	4	5	6	7	8	9	**MMM**
**HD**	9	8	7	6	5	4	3	2	1	2	3	4	5	6	7	8	9	**KO**
**HD**	9	8	7	6	5	4	3	2	1	2	3	4	5	6	7	8	9	**V**
**HD**	9	8	7	6	5	4	3	2	1	2	3	4	5	6	7	8	9	**NKE**
**HD**	9	8	7	6	5	4	3	2	1	2	3	4	5	6	7	8	9	**JPM**
**HD**	9	8	7	6	5	4	3	2	1	2	3	4	5	6	7	8	9	**VZ**
**HD**	9	8	7	6	5	4	3	2	1	2	3	4	5	6	7	8	9	**MRK**
**HD**	9	8	7	6	5	4	3	2	1	2	3	4	5	6	7	8	9	**UNH**
**HD**	9	8	7	6	5	4	3	2	1	2	3	4	5	6	7	8	9	**MSFT**

**IBM**	9	8	7	6	5	4	3	2	1	2	3	4	5	6	7	8	9	**MMM**
**IBM**	9	8	7	6	5	4	3	2	1	2	3	4	5	6	7	8	9	**KO**
**IBM**	9	8	7	6	5	4	3	2	1	2	3	4	5	6	7	8	9	**V**
**IBM**	9	8	7	6	5	4	3	2	1	2	3	4	5	6	7	8	9	**NKE**
**IBM**	9	8	7	6	5	4	3	2	1	2	3	4	5	6	7	8	9	**JPM**
**IBM**	9	8	7	6	5	4	3	2	1	2	3	4	5	6	7	8	9	**VZ**
**IBM**	9	8	7	6	5	4	3	2	1	2	3	4	5	6	7	8	9	**MRK**
**IBM**	9	8	7	6	5	4	3	2	1	2	3	4	5	6	7	8	9	**UNH**
**IBM**	9	8	7	6	5	4	3	2	1	2	3	4	5	6	7	8	9	**MSFT**

**MMM**	9	8	7	6	5	4	3	2	1	2	3	4	5	6	7	8	9	**KO**
**MMM**	9	8	7	6	5	4	3	2	1	2	3	4	5	6	7	8	9	**V**
**MMM**	9	8	7	6	5	4	3	2	1	2	3	4	5	6	7	8	9	**NKE**

**MMM**	9	8	7	6	5	4	3	2	1	2	3	4	5	6	7	8	9	**JPM**
**MMM**	9	8	7	6	5	4	3	2	1	2	3	4	5	6	7	8	9	**VZ**
**MMM**	9	8	7	6	5	4	3	2	1	2	3	4	5	6	7	8	9	**MRK**
**MMM**	9	8	7	6	5	4	3	2	1	2	3	4	5	6	7	8	9	**UNH**
**MMM**	9	8	7	6	5	4	3	2	1	2	3	4	5	6	7	8	9	**MSFT**

**KO**	9	8	7	6	5	4	3	2	1	2	3	4	5	6	7	8	9	**V**
**KO**	9	8	7	6	5	4	3	2	1	2	3	4	5	6	7	8	9	**NKE**
**KO**	9	8	7	6	5	4	3	2	1	2	3	4	5	6	7	8	9	**JPM**
**KO**	9	8	7	6	5	4	3	2	1	2	3	4	5	6	7	8	9	**VZ**
**KO**	9	8	7	6	5	4	3	2	1	2	3	4	5	6	7	8	9	**MRK**
**KO**	9	8	7	6	5	4	3	2	1	2	3	4	5	6	7	8	9	**UNH**
**KO**	9	8	7	6	5	4	3	2	1	2	3	4	5	6	7	8	9	**MSFT**

**V**	9	8	7	6	5	4	3	2	1	2	3	4	5	6	7	8	9	**NKE**
**V**	9	8	7	6	5	4	3	2	1	2	3	4	5	6	7	8	9	**JPM**
**V**	9	8	7	6	5	4	3	2	1	2	3	4	5	6	7	8	9	**VZ**
**V**	9	8	7	6	5	4	3	2	1	2	3	4	5	6	7	8	9	**MRK**
**V**	9	8	7	6	5	4	3	2	1	2	3	4	5	6	7	8	9	**UNH**
**V**	9	8	7	6	5	4	3	2	1	2	3	4	5	6	7	8	9	**MSFT**

**NKE**	9	8	7	6	5	4	3	2	1	2	3	4	5	6	7	8	9	**JPM**
**NKE**	9	8	7	6	5	4	3	2	1	2	3	4	5	6	7	8	9	**VZ**
**NKE**	9	8	7	6	5	4	3	2	1	2	3	4	5	6	7	8	9	**MRK**

**NKE**	9	8	7	6	5	4	3	2	1	2	3	4	5	6	7	8	9	**UNH**
**NKE**	9	8	7	6	5	4	3	2	1	2	3	4	5	6	7	8	9	**MSFT**

**JPM**	9	8	7	6	5	4	3	2	1	2	3	4	5	6	7	8	9	**VZ**
**JPM**	9	8	7	6	5	4	3	2	1	2	3	4	5	6	7	8	9	**MRK**
**JPM**	9	8	7	6	5	4	3	2	1	2	3	4	5	6	7	8	9	**UNH**
**JPM**	9	8	7	6	5	4	3	2	1	2	3	4	5	6	7	8	9	**MSFT**

**VZ**	9	8	7	6	5	4	3	2	1	2	3	4	5	6	7	8	9	**MRK**
**VZ**	9	8	7	6	5	4	3	2	1	2	3	4	5	6	7	8	9	**UNH**
**VZ**	9	8	7	6	5	4	3	2	1	2	3	4	5	6	7	8	9	**MSFT**

**MRK**	9	8	7	6	5	4	3	2	1	2	3	4	5	6	7	8	9	**UNH**
**MRK**	9	8	7	6	5	4	3	2	1	2	3	4	5	6	7	8	9	**MSFT**

**UNH**	9	8	7	6	5	4	3	2	1	2	3	4	5	6	7	8	9	**MSFT**

Thank you so much and wish you all the best.
