# Supplementary material for: A portfolio selection model based on the knapsack problem under uncertainty
Source: PLoS One. 2019 May 1;14(5):e0213652. doi: 10.1371/journal.pone.0213652 (PMC6493714; doi:10.1371/journal.pone.0213652)
Supplement: S3 Appendix — Interval comparison matrix. (PDF) [file pone.0213652.s011.pdf]

Table 1. An interval comparison matrix.

	S_1	S_2	S_3	S_4	S_5	S_6	S_7	S_8	S_9	S_{10}
S_1	1	[8,9]	[1,3]	[3,5]	[6,8]	[2,3]	[3,5]	[2,4]	[2,4]	[2,3]
S_2	[8,9]$^{-1}$	1	[7,9]$^{-1}$	[5,7]$^{-1}$	[2,4]$^{-1}$	[7,8]$^{-1}$	[5,7]$^{-1}$	[6,8]$^{-1}$	[7,8]$^{-1}$	[7,8]$^{-1}$
S_3	[1,3]$^{-1}$	[7,9]	1	[2,3]	[4,6]	[1,2]	[3,4]	[2,3]	[2,3]	[2,3]
S_4	[3,5]$^{-1}$	[5,7]	[2,3]$^{-1}$	1	[2,3]	[2,3]$^{-1}$	[1,1.2]	[1,1.3]$^{-1}$	[1,1.3]$^{-1}$	[1,1.4]$^{-1}$
S_5	[6,8]$^{-1}$	[2,4]	[4,6]$^{-1}$	[2,3]$^{-1}$	1	[4,6]$^{-1}$	[2,3]$^{-1}$	[3,4]$^{-1}$	[3,4]$^{-1}$	[3,5]$^{-1}$
S_6	[2,3]$^{-1}$	[7,8]	[1,2]$^{-1}$	[2,3]	[4,6]	1	[2,4]	[1,2]	[1,2]	[1,2]
S_7	[3,5]$^{-1}$	[5,7]	[3,4]$^{-1}$	[1,1.2]$^{-1}$	[2,3]	[2,4]$^{-1}$	1	[1,1.5]$^{-1}$	[1,1.5]$^{-1}$	[2,3]$^{-1}$
S_8	[2,4]$^{-1}$	[6,8]	[2,3]$^{-1}$	[1,1.3]	[3,4]	[1,2]$^{-1}$	[1,1.5]	1	[1,1.1]	[1,1.1]$^{-1}$
S_9	[2,4]$^{-1}$	[7,8]	[2,3]$^{-1}$	[1,1.3]	[3,4]	[1,2]$^{-1}$	[1,1.5]	[1,1.1]$^{-1}$	1	[1,1.2]$^{-1}$
S_{10}	[2,3]$^{-1}$	[7,8]	[2,3]$^{-1}$	[1,1.4]	[3,5]	[1,2]$^{-1}$	[2,3]	[1,1.1]	[1,1.2]	1
S_{11}	[6,8]$^{-1}$	[2,4]	[4,6]$^{-1}$	[2,3]$^{-1}$	[1,1.3]	[3,5]$^{-1}$	[2,3]$^{-1}$	[3,4]$^{-1}$	[3,4]$^{-1}$	[3,5]$^{-1}$
S_{12}	[1,2]$^{-1}$	[8,9]	[1,1.2]	[3,4]	[5,7]	[2,3]	[4,5]	[2,3]	[2,3]	[2,3]
S_{13}	[4,6]$^{-1}$	[4,6]	[3,5]$^{-1}$	[1,1.5]	[2,3]	[2,4]$^{-1}$	[1,1.1]$^{-1}$	[2,3]$^{-1}$	[2,3]$^{-1}$	[2,4]$^{-1}$
S_{14}	[2,3]$^{-1}$	[7,8]	[1,1.5]$^{-1}$	[1,1.5]	[3,5]	[1,1.2]$^{-1}$	[2,4]	[1,1.3]	[1,1.4]	[1,1.2]
S_{15}	[2,3]$^{-1}$	[7,8]	[1,1.5]$^{-1}$	[1,1.5]	[3,4]	[1,2]$^{-1}$	[2,3]	[1,1.2]	[1,1.3]	[1,1.1]
S_{16}	[5,7]$^{-1}$	[4,6]	[4,5]$^{-1}$	[1,2]$^{-1}$	[1,2]	[3,5]$^{-1}$	[1,2]$^{-1}$	[2,3]$^{-1}$	[2,3]$^{-1}$	[2,4]$^{-1}$
S_{17}	[3,5]$^{-1}$	[5,7]	[3,4]$^{-1}$	[1,1.2]$^{-1}$	[2,3]	[2,3]$^{-1}$	[1,1.1]	[1,2]$^{-1}$	[1,2]$^{-1}$	[2,3]$^{-1}$
S_{18}	[3,5]$^{-1}$	[5,7]	[3,4]$^{-1}$	[1,1.1]$^{-1}$	[2,3]	[2,4]$^{-1}$	[1,1.1]	[1,1.5]$^{-1}$	[1,1.5]$^{-1}$	[2,3]$^{-1}$
S_{19}	[1,2]$^{-1}$	[8,9]	[1,1.1]	[3,4]	[5,7]	[1,1.5]	[3,5]$^{-1}$	[2,3]	[2,3]	[2,3]
S_{20}	[2,4]$^{-1}$	[7,8]	[2,3]$^{-1}$	[2,4]	[3,5]	[1,3]$^{-1}$	[2,3]	[1,1.3]	[1,1.3]	[1,1.1]
S_{21}	[3,4]$^{-1}$	[6,8]	[2,3]$^{-1}$	[1,2]	[3,4]	[2,3]$^{-1}$	[1,2]	[1,2]$^{-1}$	[1,2]$^{-1}$	[1,2]$^{-1}$
S_{22}	[7,9]$^{-1}$	[2,3]	[5,7]$^{-1}$	[2,3]$^{-1}$	[1,1.5]$^{-1}$	[4,6]$^{-1}$	[2,3]$^{-1}$	[3,4]$^{-1}$	[3,4]$^{-1}$	[3,5]$^{-1}$
S_{23}	[2,3]$^{-1}$	[7,8]	[1,2]$^{-1}$	[2,3]	[5,6]	[1,2]$^{-1}$	[3,4]	[2,3]	[2,3]	[2,3]
S_{24}	[4,6]$^{-1}$	[4,6]	[3,4]$^{-1}$	[1,2]$^{-1}$	[2,3]	[2,4]$^{-1}$	[1,1.1]	[2,3]$^{-1}$	[2,3]$^{-1}$	[2,3]$^{-1}$
S_{25}	[7,9]$^{-1}$	[2,4]	[6,7]$^{-1}$	[3,4]$^{-1}$	[1,1.2]$^{-1}$	[4,6]$^{-1}$	[2,4]$^{-1}$	[4,5]$^{-1}$	[4,5]$^{-1}$	[4,6]$^{-1}$
S_{26}	[2,3]$^{-1}$	[7,8]	[1,2]$^{-1}$	[2,3]	[4,5]	[1,2]	[3,4]	[2,3]	[2,3]	[2,3]
S_{27}	[6,8]$^{-1}$	[3,4]	[4,6]$^{-1}$	[2,3]$^{-1}$	[1,1.1]	[3,5]$^{-1}$	[2,3]$^{-1}$	[3,4]$^{-1}$	[3,4]$^{-1}$	[3,5]$^{-1}$
S_{28}	[7,9]$^{-1}$	[2,4]	[6,8]$^{-1}$	[3,4]$^{-1}$	[1,1.2]$^{-1}$	[5,7]$^{-1}$	[2,4]$^{-1}$	[4,5]$^{-1}$	[4,5]$^{-1}$	[4,6]$^{-1}$
S_{29}	[3,5]$^{-1}$	[6,8]	[3,4]$^{-1}$	[1,1.2]	[2,3]	[2,3]$^{-1}$	[1,1.2]	[1,2]$^{-1}$	[1,2]$^{-1}$	[2,3]$^{-1}$
S_{30}	[4,6]$^{-1}$	[4,6]	[3,4]$^{-1}$	[1,2]$^{-1}$	[2,3]	[2,4]$^{-1}$	[1,2]$^{-1}$	[2,3]$^{-1}$	[2,3]$^{-1}$	[2,3]$^{-1}$

Table 1. An interval comparison matrix (continued).

	S_{11}	S_{12}	S_{13}	S_{14}	S_{15}	S_{16}	S_{17}	S_{18}	S_{19}	S_{20}
S_1	[6,8]	[1,2]	[4,6]	[2,3]	[2,3]	[5,7]	[3,5]	[3,5]	[1,2]	[2,4]
S_2	[2,4]$^{-1}$	[8,9]$^{-1}$	[4,6]$^{-1}$	[7,8]$^{-1}$	[7,8]$^{-1}$	[4,6]$^{-1}$	[5,7]$^{-1}$	[5,7]$^{-1}$	[8,9]$^{-1}$	[7,8]$^{-1}$
S_3	[4,6]	[1,1.2]$^{-1}$	[3,5]	[1,1.5]	[1,1.5]	[4,5]	[3,4]	[3,4]	[1,1.1]$^{-1}$	[2,3]
S_4	[2,3]	[3,4]$^{-1}$	[1,1.5]$^{-1}$	[1,1.5]$^{-1}$	[1,1.5]$^{-1}$	[1,2]	[1,1.2]	[1,1.1]	[3,4]$^{-1}$	[2,4]$^{-1}$
S_5	[1,1.3]$^{-1}$	[5,7]$^{-1}$	[2,3]$^{-1}$	[3,5]$^{-1}$	[3,4]$^{-1}$	[1,2]$^{-1}$	[2,3]$^{-1}$	[2,3]$^{-1}$	[5,7]$^{-1}$	[3,5]$^{-1}$
S_6	[3,5]	[2,3]$^{-1}$	[2,4]	[1,1.2]	[1,2]	[3,5]	[2,3]	[2,4]	[1,1.5]$^{-1}$	[1,3]
S_7	[2,3]	[4,5]$^{-1}$	[1,1.1]	[2,4]$^{-1}$	[2,3]$^{-1}$	[1,2]	[1,1.1]$^{-1}$	[1,1.1]$^{-1}$	[3,5]	[2,3]$^{-1}$
S_8	[3,4]	[2,3]$^{-1}$	[2,3]	[1,1.3]$^{-1}$	[1,1.2]$^{-1}$	[2,3]	[1,2]	[1,1.5]	[2,3]$^{-1}$	[1,1.3]$^{-1}$
S_9	[3,4]	[2,3]$^{-1}$	[2,3]	[1,1.4]$^{-1}$	[1,1.3]$^{-1}$	[2,3]	[1,2]	[1,1.5]	[2,3]$^{-1}$	[1,1.3]$^{-1}$
S_{10}	[3,5]	[2,3]$^{-1}$	[2,4]	[1,1.2]$^{-1}$	[1,1.1]$^{-1}$	[2,4]	[2,3]	[2,3]	[2,3]$^{-1}$	[1,1.1]$^{-1}$
S_{11}	1	[5,7]$^{-1}$	[2,3]$^{-1}$	[3,5]$^{-1}$	[3,4]$^{-1}$	[1,2]$^{-1}$	[2,3]$^{-1}$	[2,3]$^{-1}$	[5,7]$^{-1}$	[3,4]$^{-1}$
S_{12}	[5,7]	1	[4,5]	[2,3]	[2,3]	[4,5]	[3,4]	[3,4]	[1,1.1]	[2,3]
S_{13}	[2,3]	[4,5]$^{-1}$	1	[3,5]$^{-1}$	[2,4]$^{-1}$	[1,3]	[1,2]	[2,3]	[4,6]$^{-1}$	[2,3]$^{-1}$
S_{14}	[3,5]	[2,3]$^{-1}$	[3,5]	1	[1,2]	[3,4]	[2,3]	[2,3]	[2,3]$^{-1}$	[1,2]
S_{15}	[3,4]	[2,3]$^{-1}$	[2,4]	[1,2]$^{-1}$	1	[3,4]	[2,3]	[2,3]	[2,3]$^{-1}$	[1,1.2]$^{-1}$
S_{16}	[1,2]	[4,5]$^{-1}$	[1,3]$^{-1}$	[3,4]$^{-1}$	[3,4]$^{-1}$	1	[2,3]$^{-1}$	[2,3]$^{-1}$	[4,6]$^{-1}$	[3,5]$^{-1}$
S_{17}	[2,3]	[3,4]$^{-1}$	[1,2]$^{-1}$	[2,3]$^{-1}$	[2,3]$^{-1}$	[2,3]	1	[1,1.2]	[3,4]$^{-1}$	[2,4]$^{-1}$
S_{18}	[2,3]	[3,4]$^{-1}$	[2,3]$^{-1}$	[2,3]$^{-1}$	[2,3]$^{-1}$	[2,3]	[1,1.2]	1	[3,4]$^{-1}$	[2,3]$^{-1}$
S_{19}	[5,7]	[1,1.1]$^{-1}$	[4,6]	[2,3]	[2,3]	[4,6]	[3,4]	[3,4]	1	[2,3]
S_{20}	[3,4]	[2,3]$^{-1}$	[2,3]	[1,2]$^{-1}$	[1,1.2]	[3,5]	[2,4]	[2,3]	[2,3]$^{-1}$	1
S_{21}	[2,3]	[3,4]$^{-1}$	[2,3]	[2,3]$^{-1}$	[1,2]$^{-1}$	[2,3]	[1,2]	[1,2]	[3,4]$^{-1}$	[1,1.3]$^{-1}$

**S_{22}**	[1,2]$^{-1}$	[6,7]$^{-1}$	[2,4]$^{-1}$	[3,5]$^{-1}$	[3,4]$^{-1}$	[1,2]$^{-1}$	[2,3]$^{-1}$	[2,3]$^{-1}$	[6,8]$^{-1}$	[4,6]$^{-1}$
**S_{23}**	[4,5]	[2,3]$^{-1}$	[4,5]	[1,2]	[2,3]	[4,5]	[3,4]	[3,4]	[2,3]$^{-1}$	[2,3]
**S_{24}**	[2,3]	[4,5]$^{-1}$	[1,1.3]	[2,4]$^{-1}$	[2,3]$^{-1}$	[2,3]	[1,1.5]	[1,2]$^{-1}$	[4,5]$^{-1}$	[2,3]$^{-1}$
**S_{25}**	[1,1.5]$^{-1}$	[7,9]$^{-1}$	[2,4]$^{-1}$	[5,7]$^{-1}$	[4,5]$^{-1}$	[2,3]$^{-1}$	[3,4]$^{-1}$	[3,4]$^{-1}$	[7,9]$^{-1}$	[4,6]$^{-1}$
**S_{26}**	[4,5]	[2,3]$^{-1}$	[3,4]	[1,1.5]	[1,2]	[4,5]	[3,4]	[3,4]	[2,3]$^{-1}$	[2,3]
**S_{27}**	[1,1.5]$^{-1}$	[5,7]$^{-1}$	[2,3]$^{-1}$	[3,5]$^{-1}$	[3,4]$^{-1}$	[1,2]$^{-1}$	[2,3]$^{-1}$	[2,3]$^{-1}$	[5,7]$^{-1}$	[3,5]$^{-1}$
**S_{28}**	[2,3]$^{-1}$	[7,8]$^{-1}$	[2,4]$^{-1}$	[5,7]$^{-1}$	[4,5]$^{-1}$	[2,3]$^{-1}$	[3,4]$^{-1}$	[3,4]$^{-1}$	[7,8]$^{-1}$	[4,6]$^{-1}$
**S_{29}**	[2,4]	[3,4]$^{-1}$	[2,3]	[2,3]$^{-1}$	[2,3]$^{-1}$	[2,3]	[1,2]	[1,1.1]	[3,5]$^{-1}$	[2,3]$^{-1}$
**S_{30}**	[2,3]	[4,5]$^{-1}$	[1,2]$^{-1}$	[2,4]$^{-1}$	[2,3]$^{-1}$	[1,1.3]	[1,1.1]$^{-1}$	[1,2]$^{-1}$	[3,5]$^{-1}$	[2,4]$^{-1}$

Table 1. An interval comparison matrix (continued).

	**S_{21}**	**S_{22}**	**S_{23}**	**S_{24}**	**S_{25}**	**S_{26}**	**S_{27}**	**S_{28}**	**S_{29}**	**S_{30}**
**S_1**	[3,4]	[7,9]	[2,3]	[4,6]	[7,9]	[2,3]	[6,8]	[7,9]	[3,5]	[4,6]
**S_2**	[6,8]$^{-1}$	[2,3]$^{-1}$	[7,8]$^{-1}$	[4,6]$^{-1}$	[2,4]$^{-1}$	[7,8]$^{-1}$	[3,4]$^{-1}$	[2,4]$^{-1}$	[6,8]$^{-1}$	[4,6]$^{-1}$
**S_3**	[2,3]	[5,7]	[1,2]	[3,4]	[6,7]	[1,2]	[4,6]	[6,8]	[3,4]	[3,4]
**S_4**	[1,2]$^{-1}$	[2,3]	[2,3]$^{-1}$	[1,2]	[3,4]	[2,3]$^{-1}$	[2,3]	[3,4]	[1,1.2]$^{-1}$	[1,2]
**S_5**	[3,4]$^{-1}$	[1,1.5]	[5,6]$^{-1}$	[2,3]$^{-1}$	[1,1.2]	[4,5]$^{-1}$	[1,1.1]$^{-1}$	[1,1.2]	[2,3]$^{-1}$	[2,3]$^{-1}$
**S_6**	[2,3]	[4,6]	[1,2]	[2,4]	[4,6]	[1,2]$^{-1}$	[3,5]	[5,7]	[2,3]	[2,4]
**S_7**	[1,2]$^{-1}$	[2,3]	[3,4]$^{-1}$	[1,1.1]$^{-1}$	[2,4]	[3,4]$^{-1}$	[2,3]	[2,4]	[1,1.2]$^{-1}$	[1,2]
**S_8**	[1,2]	[3,4]	[2,4]$^{-1}$	[2,3]	[4,5]	[2,3]$^{-1}$	[3,4]	[4,5]	[1,2]	[2,3]
**S_9**	[1,2]	[3,4]	[2,3]$^{-1}$	[2,3]	[4,5]	[2,3]$^{-1}$	[3,4]	[4,5]	[1,2]	[2,3]
**S_{10}**	[1,2]	[3,5]	[2,3]$^{-1}$	[2,3]	[4,6]	[2,3]$^{-1}$	[3,5]	[4,6]	[2,3]	[2,3]
**S_{11}**	[2,3]$^{-1}$	[1,2]	[4,5]$^{-1}$	[2,3]$^{-1}$	[1,1.5]	[4,5]$^{-1}$	[1,1.5]	[2,3]	[2,4]$^{-1}$	[2,3]$^{-1}$
**S_{12}**	[3,4]	[6,7]	[2,3]	[4,5]	[7,9]	[2,3]	[5,7]	[7,8]	[3,4]	[4,5]
**S_{13}**	[2,3]$^{-1}$	[2,4]	[4,5]$^{-1}$	[1,1.3]	[2,4]	[3,4]$^{-1}$	[2,3]	[2,4]	[2,3]	[1,2]
**S_{14}**	[2,3]	[3,5]	[1,2]$^{-1}$	[2,3]	[5,7]	[1,1.5]$^{-1}$	[3,5]	[5,7]	[2,3]	[2,4]
**S_{15}**	[1,2]	[3,4]	[2,4]$^{-1}$	[2,3]	[4,5]	[1,2]$^{-1}$	[3,4]	[4,5]	[2,3]	[2,3]
**S_{16}**	[2,3]$^{-1}$	[1,2]	[4,5]$^{-1}$	[2,3]$^{-1}$	[2,3]	[4,5]$^{-1}$	[1,2]	[2,3]	[2,3]$^{-1}$	[1,1.3]$^{-1}$
**S_{17}**	[1,2]$^{-1}$	[2,3]	[3,4]$^{-1}$	[1,1.5]$^{-1}$	[3,4]	[3,4]$^{-1}$	[2,3]	[3,4]	[1,2]$^{-1}$	[1,1.1]
**S_{18}**	[1,2]$^{-1}$	[2,3]	[3,4]$^{-1}$	[1,2]	[3,4]	[3,4]$^{-1}$	[2,3]	[3,4]	[1,1.1]$^{-1}$	[1,2]
**S_{19}**	[3,4]	[6,8]	[2,3]	[4,5]	[7,9]	[2,3]	[5,7]	[7,8]	[3,5]	[3,5]
**S_{20}**	[1,1.3]	[4,6]	[2,3]$^{-1}$	[2,3]	[4,6]	[2,3]$^{-1}$	[3,5]	[4,6]	[2,3]	[2,4]
**S_{21}**	1	[3,5]	[2,3]$^{-1}$	[2,3]	[4,5]	[2,3]$^{-1}$	[3,4]	[4,5]	[1,2]	[2,3]
**S_{22}**	[3,5]$^{-1}$	1	[6,7]$^{-1}$	[3,4]$^{-1}$	[1,2]$^{-1}$	[6,7]$^{-1}$	[1,2]$^{-1}$	[1,2]$^{-1}$	[3,4]$^{-1}$	[2,4]$^{-1}$
**S_{23}**	[2,3]	[6,7]	1	[3,4]	[6,7]	[1,1.1]	[4,5]	[6,7]	[3,4]	[3,4]
**S_{24}**	[2,3]$^{-1}$	[3,4]	[3,4]$^{-1}$	1	[3,4]	[3,4]$^{-1}$	[2,3]	[2,4]	[1,2]$^{-1}$	[1,2]
**S_{25}**	[4,5]$^{-1}$	[1,2]	[6,7]$^{-1}$	[3,4]$^{-1}$	1	[6,7]$^{-1}$	[1,2]$^{-1}$	[1,1.1]$^{-1}$	[3,5]$^{-1}$	[3,4]$^{-1}$
**S_{26}**	[2,3]	[6,7]	[1,1.1]$^{-1}$	[3,4]	[6,7]	1	[4,5]	[6,7]	[3,4]	[3,5]
**S_{27}**	[3,4]$^{-1}$	[1,2]	[4,5]$^{-1}$	[2,3]$^{-1}$	[1,2]	[4,5]$^{-1}$	1	[1,1.2]	[2,4]$^{-1}$	[2,3]$^{-1}$
**S_{28}**	[4,5]$^{-1}$	[1,2]	[6,7]$^{-1}$	[2,4]$^{-1}$	[1,1.1]	[6,7]$^{-1}$	[1,1.2]$^{-1}$	1	[3,5]$^{-1}$	[3,4]$^{-1}$
**S_{29}**	[1,2]$^{-1}$	[3,4]	[3,4]$^{-1}$	[1,2]	[3,5]	[3,4]$^{-1}$	[2,4]	[3,5]	1	[1,2]
**S_{30}**	[2,3]$^{-1}$	[2,4]	[3,4]$^{-1}$	[1,2]$^{-1}$	[3,4]	[3,5]$^{-1}$	[2,3]	[3,4]	[1,2]$^{-1}$	1
